# De novo fatty acid synthesis by Schwann cells is essential for peripheral nervous system myelination

**DOI:** 10.1083/jcb.201706010

**Published:** 2018-04-02

**Authors:** Laura Montani, Jorge A. Pereira, Camilla Norrmén, Hartmut B.F. Pohl, Elisa Tinelli, Martin Trötzmüller, Gianluca Figlia, Penelope Dimas, Belinda von Niederhäusern, Rachel Schwager, Sebastian Jessberger, Clay F. Semenkovich, Harald C. Köfeler, Ueli Suter

**Affiliations:** 1 Institute of Molecular Health Sciences, Department of Biology, Swiss Federal Institute of Technology, ETH Zürich, Zürich, Switzerland; 2 Lipidomics Center for Medical Research, Medical University, Graz, Austria; 3 Brain Research Institute, University of Zürich, Zürich, Switzerland; 4 Division of Endocrinology, Metabolism and Lipid Research, Washington University Medical School, St. Louis, MO

## Abstract

Myelination calls for a remarkable surge in cell metabolism to facilitate lipid and membrane production. Endogenous fatty acid (FA) synthesis represents a potentially critical process in myelinating glia. Using genetically modified mice, we show that Schwann cell (SC) intrinsic activity of the enzyme essential for de novo FA synthesis, fatty acid synthase (FASN), is crucial for precise lipid composition of peripheral nerves and fundamental for the correct onset of myelination and proper myelin growth. Upon FASN depletion in SCs, epineurial adipocytes undergo lipolysis, suggestive of a compensatory role. Mechanistically, we found that a lack of FASN in SCs leads to an impairment of the peroxisome proliferator-activated receptor (PPAR) γ–regulated transcriptional program. In agreement, defects in myelination of FASN-deficient SCs could be ameliorated by treatment with the PPARγ agonist rosiglitazone ex vivo and in vivo*.* Our results reveal that FASN-driven de novo FA synthesis in SCs is mandatory for myelination and identify lipogenic activation of the PPARγ transcriptional network as a putative downstream functional mediator.

## Abstract (precis)

Montani et al. reveal that de novo fatty acid synthesis by Schwann cells, mediated by fatty acid synthase, contributes fundamentally to driving myelination in the peripheral nervous system. They identify lipogenic activation of the PPARγ transcriptional network as a putatively involved functional mechanism.

## Introduction

In the peripheral nervous system (PNS), Schwann cells (SCs) encase large-caliber axons with myelin. This essential multilamellar membrane structure allows for fast saltatory conduction of action potentials and contributes to intricate reciprocal SC-axon interactions, both central to PNS function (Pereira et al., 2012; Nave and Werner, 2014; Monk et al., 2015; Herbert and Monk, 2017). Myelination is an expensive metabolic process, demanding increased and precisely coordinated RNA and protein synthesis, epigenetic modifications, protein targeting, and massive membrane production (Taveggia et al., 2010; Nave and Werner, 2014). Notably, myelin membranes are enriched in lipids compared with other cells (Chrast et al., 2011; Nave and Werner, 2014; Schmitt et al., 2015) and possess a rather unique relative lipid composition (Chrast et al., 2011; Nave and Werner, 2014; Schmitt et al., 2015). These observations indicate that lipid synthesis and/or uptake in myelinating glial cells is likely to be tightly regulated. Nonetheless, the contribution of lipid synthesis, as opposed to uptake, and the molecular mechanisms underlying the distinctive lipid composition of myelin membranes are not clear. The mammalian target of rapamycin (mTOR) is a critical regulator of a plethora of lipogenic enzymes. In particular, mTOR complex 1 (mTORC1) appears to be important for modulating cholesterol and fatty acid (FA) synthetic pathways, including in myelinating cells (Laplante and Sabatini, 2012; Norrmén and Suter, 2013; Lebrun-Julien et al., 2014; Shimobayashi and Hall, 2014; Schmitt et al., 2015). In line with a potential critical role for overall lipogenesis, we and others have shown that mTOR (Sherman et al., 2012), mTORC1 (Norrmén et al., 2014; Figlia et al., 2017), SCAP (Verheijen et al., 2009), and the SCAP target SREBP1c (Cermenati et al., 2015) are each required for timely PNS myelin development. The functional roles of individual lipogenic enzymes and specific lipid species in this process are poorly understood (Schmitt et al., 2015). SCs lacking squalene synthase, necessary for cholesterol synthesis, exhibit reduced myelin growth (Saher et al., 2009; Schmitt et al., 2015). This phenotype recovers in adulthood, similar to findings in SCAP mutant mice (Verheijen et al., 2009; Schmitt et al., 2015). Glycolipids and phospholipids together comprise the largest proportion of myelin membrane lipids and both require FAs for their synthesis. Although various facets have been analyzed in the past, the functional role of endogenous FA synthesis in myelinating glia has not been addressed. Cells mostly derive FAs from dietary sources (Currie et al., 2013). However, most FAs, referred to as nonessential FAs, can also be synthesized intracellularly. Fatty acid synthase (FASN) is responsible for the catalysis of all seven steps leading to the synthesis of 16-carbon palmitic acid, beginning with the condensation of acetyl–coenzyme A and malonyl–coenzyme A. Palmitic acid is the substrate for the synthesis of more complex nonessential FAs (Currie et al., 2013). Under increased lipid demand, e.g., a high proliferative rate, cells are capable of up-regulating FASN. Thus, both progenitor (Knobloch et al., 2013) and cancer (Menendez and Lupu, 2007; Currie et al., 2013) cells rely on FASN activity for proliferation, and FASN inhibitors are currently under study as potential anticancer therapies (Menendez and Lupu, 2007; Currie et al., 2013). Notably, FAs can be used for purposes beyond membrane lipid production. They can also affect gene transcription in a tissue-specific manner via modulation of the activity of nuclear peroxisome proliferator-activated receptors (PPARα, β, and γ; Ahmadian et al., 2013).

Because of their high rates of membrane production, the high lipid content of their membranes, and their peculiar relative lipid composition, we hypothesized that SCs rely critically on endogenous FA synthesis during development, rather than solely on FA uptake. Indeed, both FASN and PPARγ are expressed in myelinating SCs (Verheijen et al., 2003; Yamagishi et al., 2008; Cao et al., 2012; Nave and Werner, 2014), and the level of FASN expression in the PNS is temporally linked to the myelination process (Salles et al., 2002). These observations prompted us to investigate the specific role of FASN-mediated de novo FA synthesis in SC myelination. To this end, we examined the functional consequences of conditional depletion of FASN in SCs in vivo, in conjunction with molecular mechanisms associated with this alteration. Our data identified FASN-mediated FA synthesis in SCs as a fundamental prerequisite for correct myelination. Moreover, we found that lipogenic activation of the PPARγ transcriptional network is a putative contributor to this process.

## Results

### De novo FA synthesis in SCs is required for fully functional PNS development

**Figure 1.**
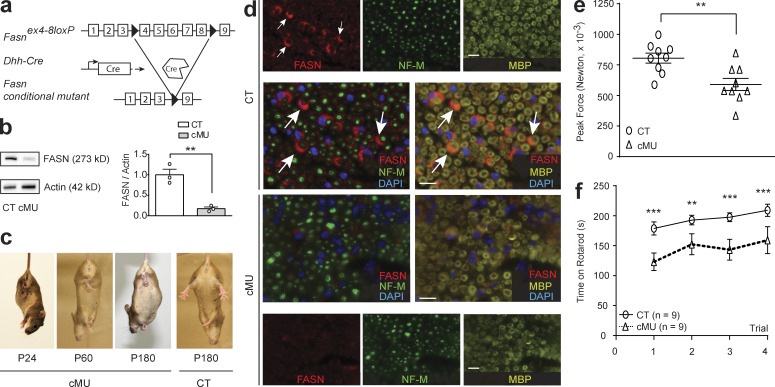
**De novo FA synthesis in SCs is required for correct PNS function. (a)** Conditional *Fasn* allele inactivation in vivo upon *Dhh*-driven Cre expression. **(b)** FASN immunoblot and quantification graph, normalized to β-actin, of protein lysates of sciatic nerves (P40). *n* = 3 blots, each data point represents an independent experiment with an independent set of lysates from three control (CT) and three conditional mutant (cMU) mice (unpaired two-tailed two sample Student’s *t* test, P = 0.0044, *t* = 5.816); **, P < 0.01. **(c)** Aberrant hindlimb clasping in cMU mice by tail lift. **(d)** Representative immunostaining of cross sectioned sciatic nerves from P14 CT and cMU mice; *n* = 3 mice for each, CT and cMU. Note the prominent cytoplasmic FASN expression in SCs of nerves of CT but not cMU mice. Myelin marker: myelin basic protein, MBP; axonal marker: neurofilament M, NF-M; nuclear marker: DAPI. Bars, 10 µm. **(e and f)** P40 cMU mice showed reduced grip strength (unpaired two-tailed two sample Student’s *t* test, P = 0.0044, *t* = 3.310); **, P < 0.01, and decreased latency to fall from a Rotarod (two-way ANOVA, trials P *=* 0.0333*, F*_3,24_ = 3.424, Genotype P = 0.01, *F*_1,8_ = 11.25, with Bonferroni’s multiple comparisons test, trial 1 P < 0.0001, *t* = 5.284; trial 2 P = 0.0033, *t* = 3.819; trial 3 P = 0.0001, *t* = 5.140; trial 4 P = 0.0003, *t* = 4.747); **, P < 0.01; ***, P < 0.001, compared with CT. Data points represent *n* = 9 mice for each, CT and cMU. Bars represent mean ± SEM.

We first addressed the role of de novo FA synthesis in PNS myelination by conditionally ablating *Fasn* in SCs in vivo (called mutant hereafter). Mice carrying floxed *Fasn* alleles (Chakravarthy et al., 2005) were crossed with mice expressing Cre recombinase under the control of *Desert hedgehog* (*Dhh*) gene regulatory sequences (Jaegle et al., 2003; Fig. 1 a). Loss of FASN expression in SCs of sciatic nerves of mutant mice was confirmed by immunoblotting (Fig. 1 b) and immunohistochemistry (Fig. 1 d). Mutant mice were viable and fertile, with litters born according to expected Mendelian distributions. However, qualitative phenotypic analysis revealed abnormal hindlimb clasping upon tail-lift (Fig. 1 c). In parallel, we found that mutants displayed moderately decreased forelimb muscular peak force in a grip-strength test (Fig. 1 e) and decreased latency to fall from an accelerating Rotarod compared with controls (Fig. 1 f). Together, these results are consistent with a requirement of accurate FASN activity in SCs to achieve complete PNS function during development.

### FA synthesis is largely dispensable for SC survival and proliferation

During early postnatal development, SCs undergo extensive proliferation. Reaching a threshold number is thought to be a limiting factor for successful SC-axon sorting, i.e., the process by which SCs segregate out large-caliber axons from axonal bundles into a one-to-one (SC-to-axon) promyelinating relationship, the developmental step preceding onset of myelination (Benninger et al., 2007; Feltri et al., 2016). Because synthesis of FAs is critical for other highly proliferative cells, we analyzed SC proliferation and survival at birth and postnatal day 5 (P5). However, we found no detectable differences between mutants and controls (Fig. S1, a and b). As such, we conclude that FASN and de novo FA synthesis are not required for SC proliferation and survival in early postnatal PNS development.

### De novo FA synthesis in SCs is essential for the correct onset of PNS myelination

**Figure 2.**
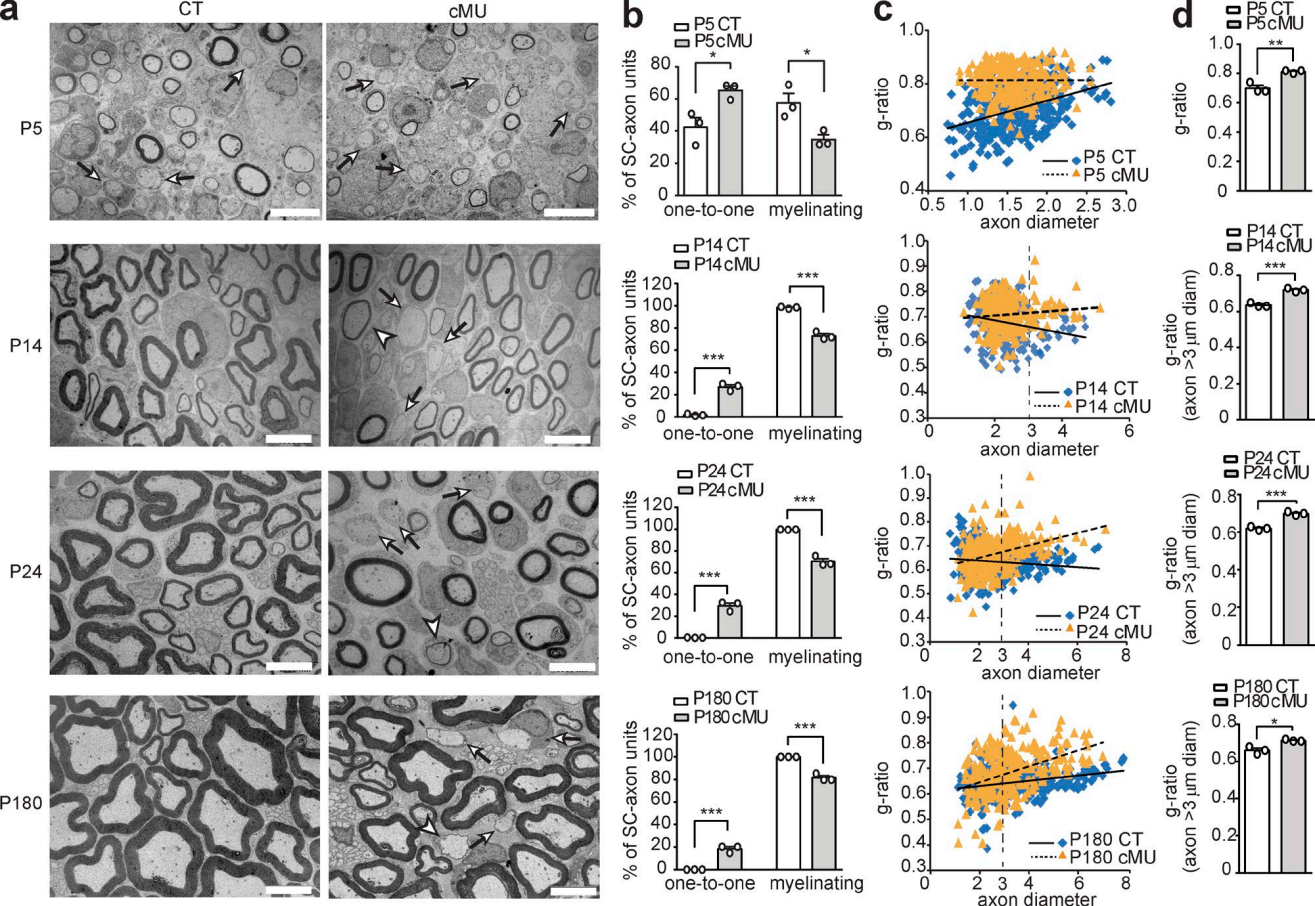
**De novo FA synthesis in nerve SCs is essential to achieve proper PNS myelination. (a)** Representative EM images of mutant (cMU) and control (CT) sciatic nerve cross sections. Whereas in CT nerves individual large-caliber axons are sorted by SCs to the promyelinating stage (examples indicated by arrows) and progressively myelinated, many SCs remain blocked at promyelination in cMU (examples indicated by arrows). cMU nerves also display more thinly myelinated axons (examples indicated by arrowheads). Bars, 5 µm. **(b)** Corresponding graphs with quantification of percentage of SC-axon units at the one-to-one versus myelinating stage. Data points represent *n* = 3 mice for each, CT and cMU, with at least 250 sorted axons quantified in one nerve per animal (unpaired two-tailed two sample Student’s *t* test, P5 cMU vs. CT, P = 0.0249, *t* = 3.50; P14 cMU vs. CT, P = 0.0002, *t* = 12.65; P24 cMU vs. CT, P = 0.0004, *t* = 11.29; and P180 cMU vs. CT, P = 0.0006, *t* = 9.859); *, P < 0.05; ***, P < 0.001. **(c)** Linear correlation of g-ratio versus axon diameter in sciatic nerves of cMU compared with CT. At least 80 myelinated axons per mouse analyzed, three mice for each, CT and cMU. **(d)** Overall hypomyelination at P5 and of grouped larger (≥3 µm in diameter) axons at later stages (P14, P24, and P180) in cMU, as shown by g-ratio analysis. Data points represent *n* = 3 mice for each, CT and cMU (unpaired two-tailed two sample Student’s *t* test, P5 P = 0.0058, *t* = 5.376; P14 P = 0.0007, *t* = 9.449; P24 P = 0.0006, *t* = 9.798; and P180 P = 0.0114, *t* = 4.438); *, P < 0.05; **, P < 0.01; ***, P < 0.001. Bars represent mean ± SEM.

To characterize whether FASN is required at later stages of SC development and/or for myelination, we analyzed the ultrastructural morphology of mutant and control sciatic nerves by comparative EM. Consistent with the unaltered SC proliferation and survival, mutants showed no overt defects in axonal segregation. Instead, we found a major impairment in myelination onset and mild effects on myelin growth (Fig. 2). In P5 control nerves, 57.59 ± 5.84% of sorted SC-axon units had started myelination, whereas only 34.80 ± 2.88% had progressed to this stage in mutants (Fig. 2, a and b). In addition, mutant myelinating SCs showed overall thinner myelin compared with controls. Quantification by g-ratio analysis (a conventional measure of myelin thickness, i.e., axon diameter/[axon + myelin] diameter) confirmed substantial hypomyelination (Fig. 2, c and d). As development of sciatic nerves progressed, practically all large-caliber axons became myelinated in controls at P14. In contrast, ∼25% of sorted SC-axon units remained arrested at the promyelinating stage in P14 mutant nerves (27.02 ± 1.94% in mutants vs. 1.69 ± 0.51% in controls; Fig. 2 a [arrows] and b). Hypomyelination was still present (Fig. 2, a [arrowheads] and c), but a significant increase in the mean g-ratio was found only if axons >3 µm in diameter were analyzed as a group (Fig. 2 d). These features remained stable in adolescent (P24) and adult (P180; Fig. 2, a–d) control and mutant nerves, confirming a major block at the onset of myelination in a subset of myelination-competent SCs, together with consistent, albeit mild, hypomyelination. To corroborate the morphological data, we examined the expression of the octamer-binding transcription factor Oct6 that is normally present at high levels in promyelinating SCs, but down-regulated once the cells shift toward a myelinating phenotype (Jessen and Mirsky, 2005). Indeed, we found increased amounts of Oct6 in protein lysates from sciatic nerves of P40 mutants compared with controls by immunoblotting (Fig. S1 c). We conclude that SC-intrinsic FASN plays multiple important roles in nerve development, including a striking effect on the onset of myelination.

### De novo FA synthesis in SCs is not majorly important for axonal maintenance

Next, we examined whether FA synthesis in SCs contributes to myelin-supported axonal maintenance. Although we observed, at a very low frequency, some stressed axons presumably compressed because of myelin vacuolization and/or splitting in mutants (Fig. S1, d and e), the total number of sorted axons (i.e., at the myelinating or promyelinating stage) per P180 sciatic nerve cross section did not differ between controls and mutants (Fig. S1, f). Our findings indicate that FASN activity in SCs is not crucial for the maintenance of sorted axons, at least until P180.

### SCs in nerve roots are more strongly reliant upon de novo FA synthesis than SCs in distal nerves

**Figure 3.**
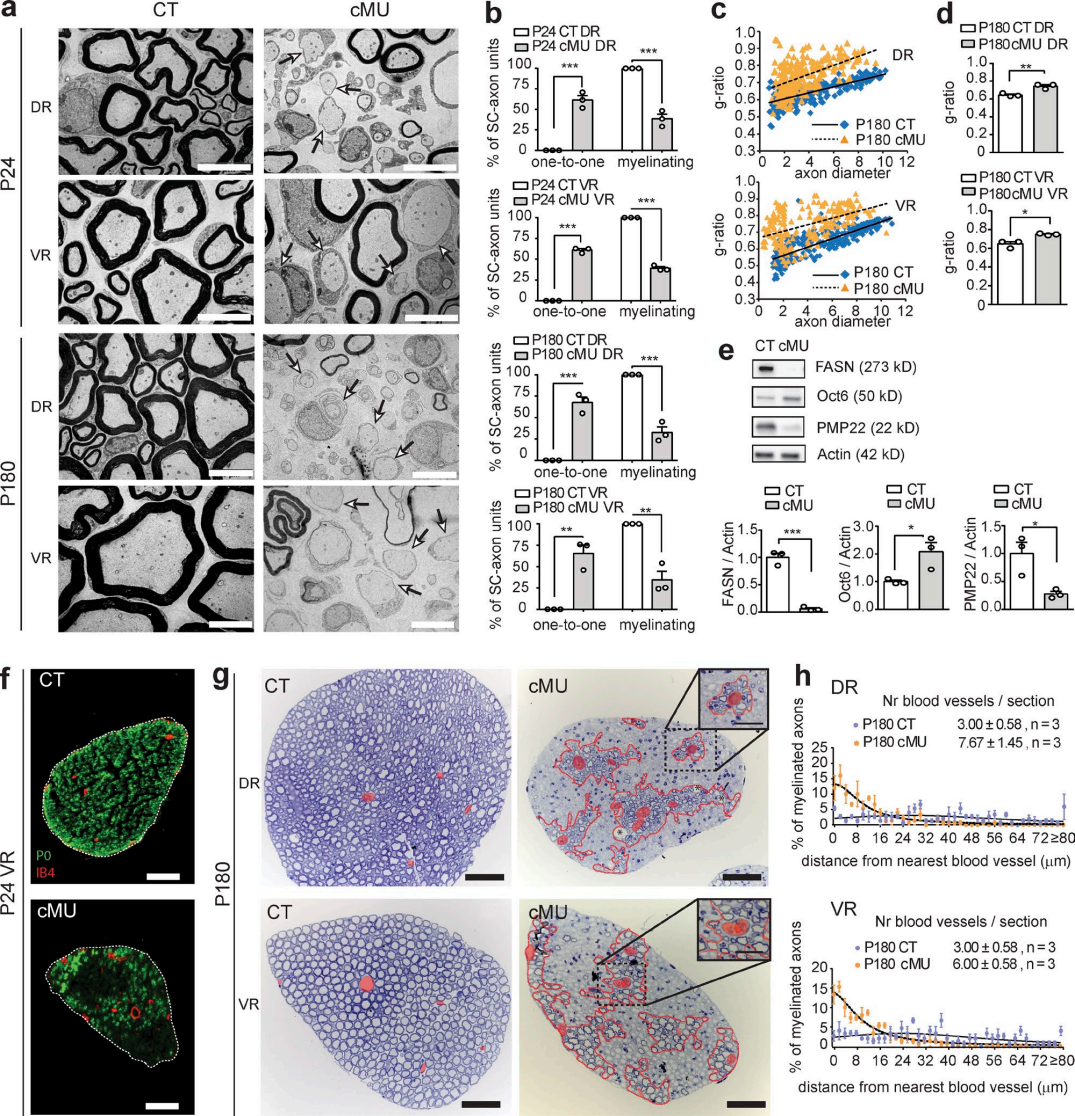
**SCs in nerve roots are strongly dependent on de novo FA synthesis. (a)** Exemplary EM images of lumbar dorsal root (DR) and ventral root (VR) cross sections from mutant (cMU) and (CT) mice. In cMU roots, many SC-axon units remain blocked at the promyelinating stage (examples indicated by arrows). Bars, 5 µm. **(b)** Corresponding graphs depicting complete quantification on fully reconstructed, EM-recorded root cross sections of the percentage of SC-axon units at the one-to-one versus myelinating stage. Data points represent *n* = 3 mice for each, CT and cMU (unpaired two-tailed two sample Student’s *t* test, P24 DR cMU vs. CT P = 0.0004, *t* = 11.10; P24 VR cMU vs. CT P < 0.0001, *t* = 32.94; P180 DR cMU vs. CT P = 0.0005, *t* = 10.39; and P180 VR cMU vs. CT P = 0.0026, *t* = 6.694); **, P < 0.01; ***, P < 0.001. **(c)** Correlation of g-ratio versus axon diameter. Graphs show values obtained from at least 40 myelinated axons per root per mouse; *n* = 3 mice for each, CT and cMU. **(d)** Hypomyelination shown by g-ratio analysis. Data points represent *n* = 3 mice for each, CT and cMU (unpaired two-tailed two sample Student’s *t* test, P180 DR P = 0.0093, *t* = 4.704; P180 VR P = 0.0132, *t* = 4.243); **, P < 0.01; *, P < 0.05. **(e)** Immunoblots normalized to β-actin and corresponding graphs. *n* = 3 blots, each data point representing an independent experiment with an independent set of lysates from pooled DRs and VRs from three CT and three cMU mice (unpaired two-tailed two sample Student’s *t* test, FASN, P = 0.0003, *t* = 12.04; Oct6, P = 0.0328, *t* = 3.204; PMP22, P = 0.0250, *t* = 3.496); *, P < 0.05; ***, P < 0.001. **(f)** Representative immunohistochemistry of VRs of P24 CT and cMU showing myelinating SCs expressing myelin Protein Zero (P0). In cMU, myelinating axons are enriched in proximity to IB4-positive blood vessels. Bars, 50 µm. *n* = 3 mice for each, CT and cMU. **(g)** Representative toluidine-blue stained semithin cross sections of DRs and VRs (P180). In cMU, the areas covered by myelinated axons (red dotted line) are stereotypically positioned around blood vessels (false-colored in red). Bars, 50 µm; (insets) 25 µm. *n* = 3 mice for each, CT and cMU. **(h)** Graphs with relative distribution of myelinated axons, categorized by their distance from the closest blood vessel, in DRs and VRs (P180), CT and cMU. *n* = 3 mice for each, CT and cMU. Number (Nr) of blood vessels per toluidine blue–stained nerve cross section; mean ± SEM; *n* = 3 mice for each, CT and cMU (unpaired two-tailed two sample Student’s *t* test, DR, P = 0.0405, *t* = 2.985; VR, P = 0.0213, *t* = 3.674). Bars represent mean ± SEM.

SCs in distal nerves are directly derived from neural crest precursors, whereas SCs located in the nerve roots are derived in large part from boundary cap cells (Maro et al., 2004; Aquino et al., 2006; Jacob, 2015). Thus, we asked whether SCs located in the roots would show a similar dependence upon FA synthesis as observed in distal nerves. First, we analyzed the ultrastructure of dorsal (sensory) lumbar roots (DRs) and ventral (motor) lumbar roots (VRs) at P24 and P180 by EM. Roots from control mice displayed extensive myelination at P24 and P180 (Fig. 3 a). In mutants, however, both DRs and VRs contained many sorted SC-axon units aberrantly arrested at the promyelinating stage. Quantification revealed 60–70% of sorted SC-axon units affected in this way (in P24 DRs: 61.38 ± 5.53% in mutants vs. 0.0 ± 0.0% in controls; in P24 VRs: 60.86 ± 1.85% in mutants vs. 0.0 ± 0.0% in controls; in P180 DRs: 67.34 ± 6.48% in mutants vs. 0.0 ± 0.0% in controls; and in P180 VRs: 65.49 ± 9.79% in mutants vs. 0.0 ± 0.0% in controls; Fig. 3, a [arrows] and b). Consistent with these morphological findings, immunoblotting revealed increased expression of Oct6 and reduced expression of the PNS myelin protein PMP22 in protein lysates of roots of P40 mutants compared with control mice (Fig. 3 e). Moreover, the myelinated fibers present in the mutant roots were hypomyelinated (Fig. 3, c and d), but we did not observe obvious signs of potential de- and remyelination. Interestingly, these myelinated fibers were stereotypically positioned, framing an increased number of nerve blood vessels (Fig. 3, f–h), consistent with a possible compensatory role for uptake of FA from blood.

**Figure 4.**
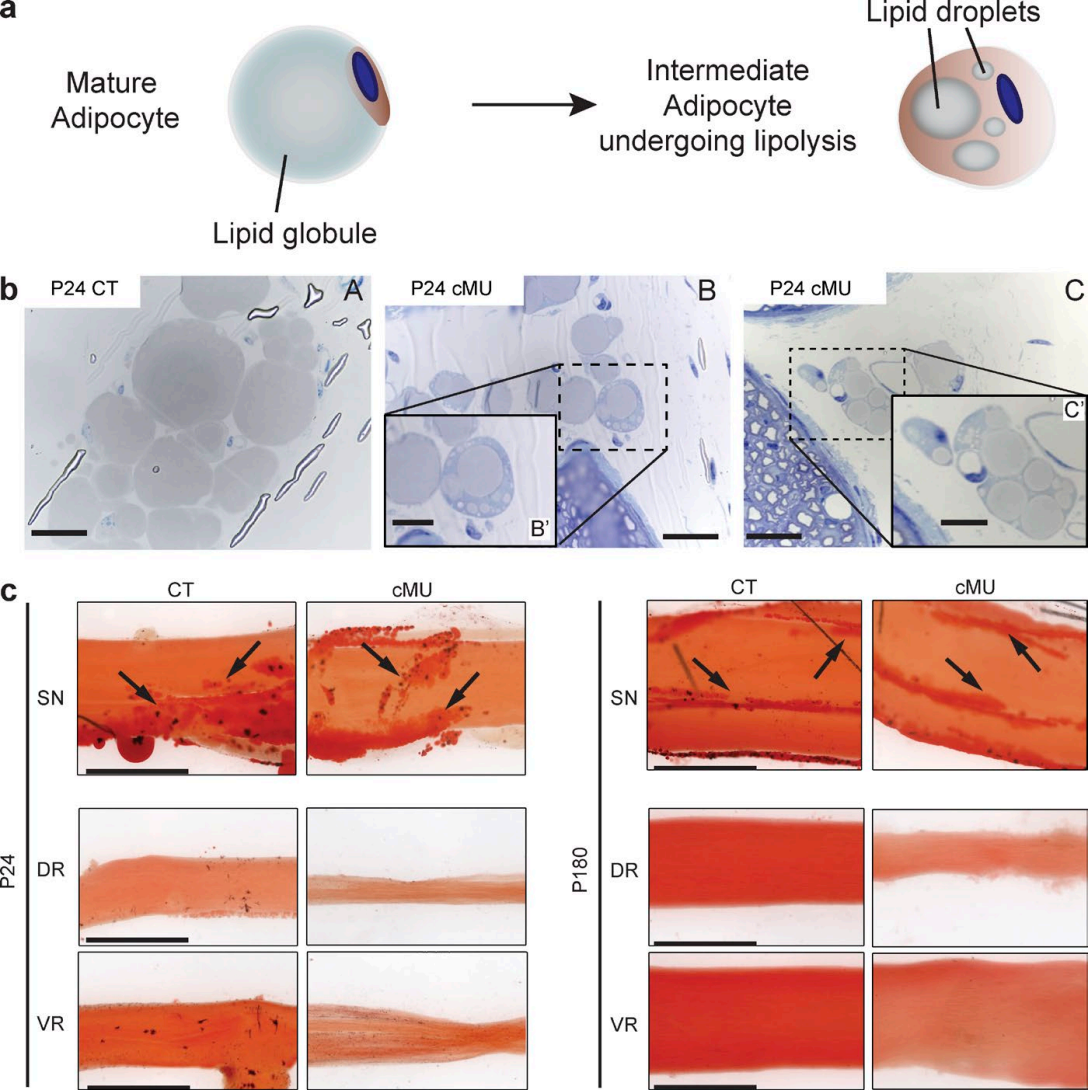
**Epineurial adipocytes may partially sustain myelination in the absence of SC FA synthesis. (a)** Schematic of morphological changes in adipocytes during lipolysis. **(b)** Representative toluidine-stained cross sections of sciatic nerves depicting mature epineurial adipocytes in control mice (CT; A) and adipocytes with multiple lipid droplets in mutant mice (cMU; B and inset B′, and C and inset C′). Bars: (B and C) 20 µm; (B′ and C′, insets) 10 µm. *n* = 3 mice for each, CT and cMU. **(c)** Oil Red O staining of sciatic nerves (SNs), DRs, and VRs from P24 and P180 CT and cMU confirmed the presence of epineurial adipocytes (examples indicated by arrows) in nerves, while revealing their absence in roots. Depicted images are representative of observed samples, *n* = 3 mice for each CT and cMU. Bars, 500 µm.

The more pronounced phenotype in the nerve roots compared with distal nerves might be ascribed to different metabolic demands of SCs and/or to the presence of diverse compensating mechanisms. The latter prompted us to examine the nerve epineurium in which adipocytes accumulate after birth (Nadra et al., 2012). We found that these cells displayed a mature morphology in sciatic nerves of control mice (P24), with a single large lipid globule (Cristancho and Lazar, 2011; Rosen and Spiegelman, 2014; Fig. 4, a and b). In contrast, P24 mutant nerves contained numerous epineurial adipocytes with multiple lipid droplets surrounded by cytoplasm (Fig. 4, a and b), a characteristic of adipocytes undergoing lipolysis (Cristancho and Lazar, 2011; Rosen and Spiegelman, 2014). Oil-Red-O staining of epineuria of P24 and P180 sciatic nerves of controls and mutants was consistent with the presence of large numbers of adipocytes (Fig. 4 c, arrows). Interestingly, however, both VRs and DRs, in controls and mutants, lacked such strong staining, suggesting a lack of adipocytes (Fig. 4 c). Given that epineurial adipocytes have no major critical role in myelin formation under normal conditions (Nadra et al., 2012), one interpretation of these results is that epineurial adipocytes may be able to support myelination in distal nerves in the absence of de novo FA synthesis as a source of supplementary lipids.

Our results show that SCs in nerve roots are more dependent on de novo FA synthesis than SCs in distal nerves. Because DRs and VRs were affected similarly, the findings also suggest that myelination of distal sensory and motor fibers are comparably reliant on FASN activity. In agreement, saphenous (mainly sensory) and quadricep (motor-enriched) nerves shared a comparable phenotype among each other (Fig. S2).

### De novo FA synthesis in SCs is required for accurate lipid composition of peripheral nerves

**Figure 5.**
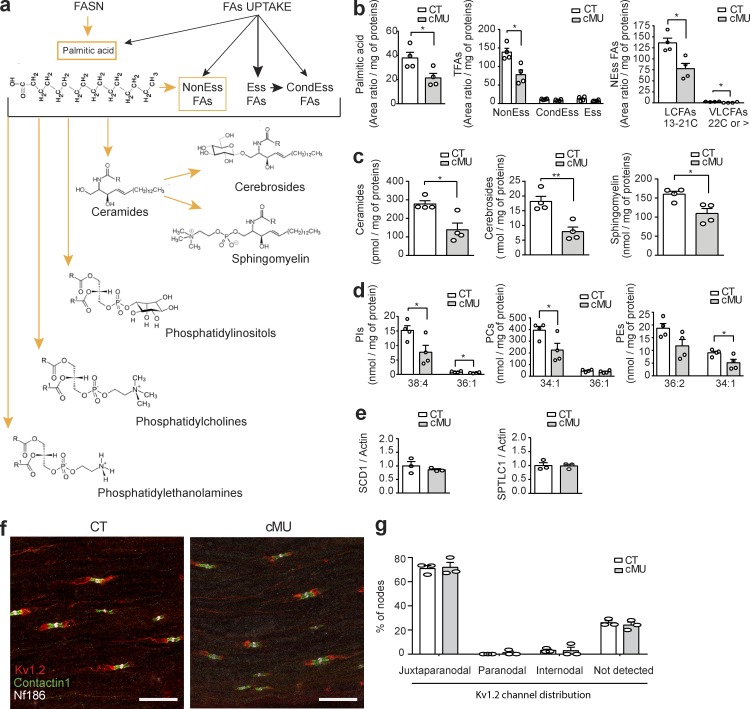
**De novo FA synthesis in SCs is crucial for correct lipid composition of peripheral nerves. (a)** Proposed hypothetical schema of sources of FAs and derived lipids in nerves. Proper nerve pools of nonessential (NonEss) FAs and derived complex lipids rely on endogenous FA synthesis in SCs (highlighted in orange), in contrast to essential (Ess) FAs, taken up through the diet, and conditional essential (CondEss) FAs, taken up through the diet or synthesized from essential FAs. **(b)** Reduction in palmitic acid and nonessential FAs (NEss), both long-chain (LCFAs) and very-long-chain (VLCFAs), in nerves of P24 mutant (cMU) mice compared with controls (CT; unpaired two-tailed two sample Student’s *t* test, palmitic acid P = 0.0313, *t* = 2.796; NEss FAs P = 0.0106, *t* = 3.655; CondEss FAs P = 0.0640, *t* = 2.266; Ess FAs P = 0.1292, *t* = 1.758; LCFAs P = 0.0114, *t* = 3.597, VLCFAs P = 0.0208, *t* = 3.111); *, P < 0.05. **(c)** Content of ceramides, cerebrosides, and sphingomyelin in nerves of P24 mutant (cMU) mice compared with controls (CT; unpaired two-tailed two sample Student’s *t* test, ceramides P = 0.0133, *t* = 3.471; cerebrosides P = 0.0045, *t* = 4.417; sphingomyelin P = 0.0157, *t* = 3.334); *, P < 0.05; **, P < 0.01. **(d)** Quantification of the two most abundant species of phosphatidylinositols (PIs), phosphatidylcholines (PCs), and phosphatidylethanolamines (PEs) in nerves of P24 cMU compared with CT (unpaired two-tailed two sample Student’s *t* test, PI 38:4 P = 0.0416, *t* = 2.583; PI 36:1 P = 0.0355, *t* = 2.701; PC 34:1 P = 0.0419, *t* = 2.577; PC 36:1 P = 0.2728, *t* = 1.207; PE 36:2 P = 0.0702, *t* = 2.199; PE 34:1 P = 0.0414, *t* = 2.587); *, P < 0.05. Data points represent *n* = 4 mice for each, CT and cMU, in entire lipidomic analysis. Lipid amounts normalized to the total protein contents of the sample (two pooled nerves per mouse). **(e)** Graphs of qRT-PCR analysis of SCD1 and SPTLC1 in sciatic nerves of P40 CT and cMU mice. qRT-PCR data normalized to β-actin, data points represent *n* = 3 mice for each, CT and cMU mice (unpaired two-tailed two sample Student’s *t* test, SCD1 P = 0.4292, *t* = 0.8786; SPTLC1 P = 0.9143, *t* = 0.1146). **(f)** Representative z-projections of stacks imaged by confocal microscopy of immunostaining of the juxtaparanodal potassium channel Kv1.2, the paranodal contactin 1, and the nodal neurofascin 186 proteins, in longitudinal sections of sciatic nerves from P30 CT and cMU mice, *n* = 3 mice for each, CT and cMU. Bar, 20 µm. **(g)** Graph showing quantification of Kv1.2 potassium channel localization, as the percentage of nodes with expression restricted to the juxtaparanode (juxtaparanodal), or aberrantly present in the paranode (paranodal), diffuse in the internode (internodal), or not detected. The graph represents values obtained from at least 40 nodes per mouse, from three mice for each, CT and cMU (two-way ANOVA, genotype P = 0.3739; *F_1,4_* = 1, localization P < 0.0001; *F_3,12_* = 312.6, with Sidak’s multiple comparisons test, cMU vs. CT juxtaparanodal P = 0.9966, *t* = 0.312; cMU vs. CT paranodal P = 0.9939, *t* = 0.3638; cMU vs. CT internodal P > 0.9999, *t* = 0.0539; cMU vs. CT not detected P = 0.9565, *t* = 0.6209). Bars represent mean ± SEM.

After establishing that FASN activity in SCs is crucial for proper myelination in vivo, we explored how SC-intrinsic de novo FA synthesis contributes to the lipid composition of nerves. We performed a lipidomic analysis to compare FAs and FA-derived lipid classes of sciatic nerves dissected from P24 FASN mutant and control mice, after carefully peeling off the epineurium to avoid adipocyte contamination (Fig. 5). These studies revealed a strong reduction in the content of palmitate, the direct product of FASN enzymatic activity, in mutants compared with controls (area ratio per milligram of proteins: 21.49 ± 3.67 in mutants vs. 37.95 ± 4.61 in controls; Fig. 5 b). Furthermore, the total amount of nonessential FAs was robustly decreased in mutant nerves (area ratio per milligram of proteins: 78.30 ± 12.64 in mutants vs. 138.8 ± 10.68 in controls; Fig. 5 b and Fig. S3), because of reductions in long-chain and very-long-chain nonessential FAs (Fig. 5 b and Fig. S3). No significant changes were detectable in the total amount of essential FAs, derived from diet, and conditional-essential FAs (Fig. 5 b and Fig. S3). We also found that loss of SC-intrinsic FASN activity affected the levels of more complex FA-derived lipid species (Fig. 5, c and d; and Fig. S3). Sciatic nerves of mutants displayed a substantially reduced content of ceramides (Fig. 5 c and Fig. S3), and the ceramide derivatives sphingomyelin and cerebrosides (Fig. 5 c and Fig. S3). Mutant mice with defective cerebroside synthesis have been shown to display altered PNS nodes of Ranvier, including pronounced potassium channel mislocalization shifting from juxtaparanodal to paranodal domains, accompanied by nerve conduction abnormalities (Bosio et al., 1996; Dupree et al., 1998, 1999). However, we did not detect mislocalization of Kv1.2 potassium channels in sciatic nerves of FASN mutant mice (Fig. 5, f and g).

The highly abundant phosphatidylinositol 38:4, phosphatidylcholine 34:1, and phosphatidylethanolamine 34:1 species were also reduced in nerves of FASN mutant mice, whereas the most abundant phosphatidylethanolamine, 36:2, showed a trend toward reduction (Fig. 5 d and Fig. S3). The observed lipidomic changes occurred without significant alterations in transcript levels of other key enzymes for FAs and derived lipid metabolism, i.e., stearoyl–coenzyme A desaturase (SCD1), the rate limiting enzyme for the synthesis of unsaturated FAs, and serine palmitoyl transferase long-chain subunit 1 (SPTLC1), a central enzyme in the synthesis of sphingolipids (Fig. 5 e). Moreover, we observed no significant accumulation of single lipid species (Fig. S3). These data establish that SC-intrinsic de novo FA synthesis is essential to build up proper levels of palmitate, nonessential FAs, and complex lipids in peripheral nerves (Fig. 5 a).

### Increased lipid dietary intake does not substitute for endogenous SC FA synthesis

**Figure 6.**
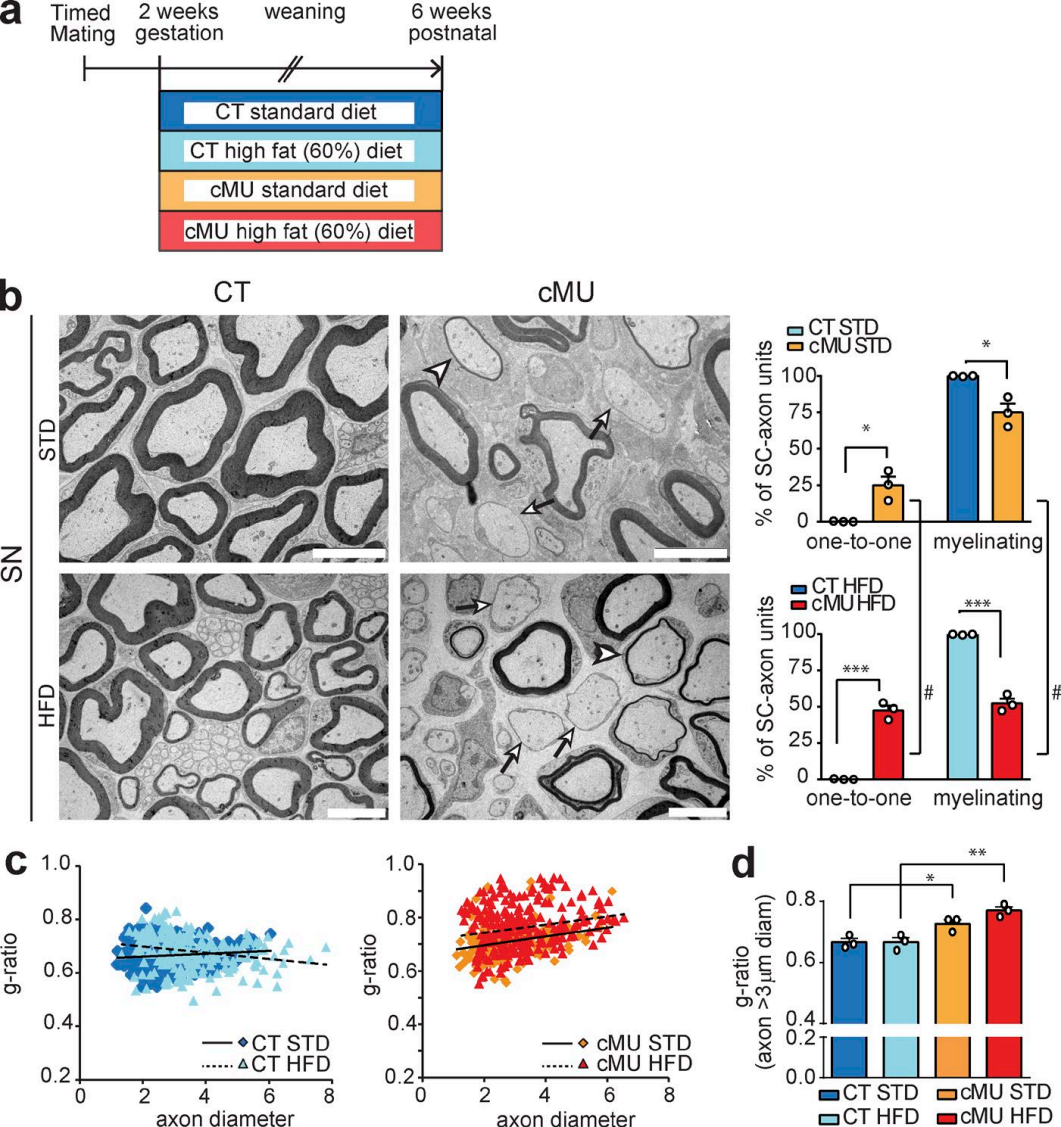
**Increased lipid dietary intake did not substitute for SC endogenous FA synthesis. (a)** Schema depicting the experimental design. **(b)** Exemplary EM micrographs of sciatic nerve cross sections from mutant (cMU) and control (CT) mice fed an STD or HFD, and graphs of the percentage of one-to-one versus myelinating SC-axon units. Sciatic nerves (SNs) from cMU mice fed a standard or HFD showed SCs blocked at the promyelinating stage (examples indicated by arrows) and hypomyelinated axons (examples indicated by arrowheads). Bars, 5 µm. Data points represent *n* = 3 mice for each, CT and cMU, and for each, STD and HFD (unpaired two-tailed two sample Student’s *t* test, STD cMU vs. CT, P = 0.0133, *t* = 4.240; HFD cMU vs. CT, P = 0.0002, *t* = 13.98; cMU HFD vs. STD, P = 0.0290, *t* = 3.334); ^#^, P < 0.05; *, P < 0.05; ***, P < 0.001. ^#^, Significance of high-fat compared with STD. *, Significance of cMU compared with CT. **(c)** Linear correlation of g-ratio versus axon diameter. The graph represents values obtained from at least 50 myelinated axons per mouse, from three mice for each, CT and cMU. **(d)** g-ratio analysis of larger (≥3 µm in diameter) axons shows no detectable amelioration of sciatic nerve hypomyelination of cMU mice fed an HFD. Data points represent *n* = 3 mice for each, CT and cMU, and for each, standard and HFD (one-way ANOVA, treatment P = 0.0011, *F_3,8_* = 15.22, with Sidak’s multiple comparisons test; cMU STD vs. CT STD P = 0.0436, *t* = 3.286; cMU HFD vs. CT HFD P = 0.0019, *t* = 5.660; CT HFD vs. CT STD P > 0.9999, *t* = 0.0; cMU HFD vs. cMU STD P = 0.1682, *t* = 2.373); *, P < 0.05; **, P < 0.01. Bars represent mean ± SEM.

We next tested whether a lack of endogenous FA synthesis in SCs could be rescued by increasing dietary lipid intake. Thus, we fed mutant and control mice a high-fat diet (HFD; 60% caloric intake from fat and enriched in palmitic acid; complete list of FA contents is shown in Table S1) from embryonic day 14 to 6 wk of age, according to an established protocol capable of rescuing defective astrocytic lipid synthesis in vivo (Camargo et al., 2012; Fig. 6 a). This dietary intervention did not ameliorate the phenotype observed in nerves of FASN mutant mice (Fig. 6, b and c). On the contrary, mutants fed a HFD had a more pronounced phenotype (Fig. 6 b). Comparable to our previous experiments (Fig. 2, a and b), mutants fed a standard diet (STD) displayed 25.0 ± 5.86% of sciatic nerve SCs aberrantly blocked at the promyelinating stage (Fig. 6 b). This percentage was increased to 47.56 ± 3.39 in mutants fed a HFD (Fig. 6 b). The effect is unlikely to be ascribed to intrinsic detrimental effects of the diet per se, as we detected no change in nerves of control mice fed with a high-fat compared with an STD (Fig. 6 b and c). Rather it appears that nerves lacking SC-intrinsic FASN activity are uniquely susceptible to high-fat feeding. The HFD protocol also did not rescue hypomyelination of larger (≥3 µm in diameter) axons in sciatic nerves of mutants compared with controls (Fig. 6 d). These results indicate that SC myelination relies on endogenous synthesis of FAs. This dependence cannot be circumvented by high dietary fats under the conditions that we applied.

### FA synthesis in SCs triggers activation of the PPARγ transcriptional program

**Figure 7.**
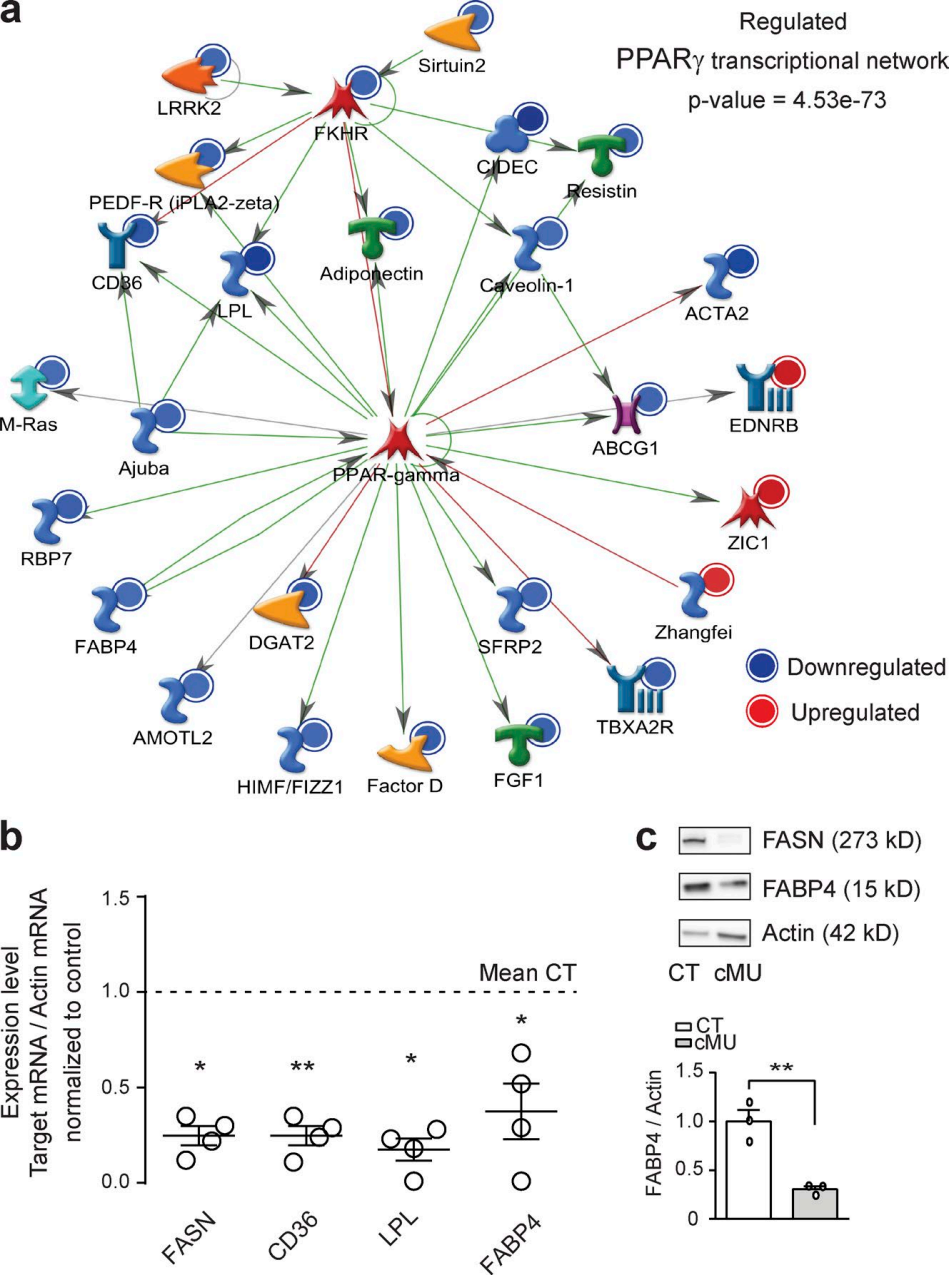
**FA synthesis in SCs triggers the activation of the PPARγ transcriptional program. (a)** MetaCore analysis including all identified regulated transcripts (ANOVA with Benjamini-Hochberg false discovery rate for multiple testing correction, P < 0.05, ≥1.5-fold change) in sciatic nerves of mutant (cMU) mice compared with controls (CT) revealed inhibition of the PPARγ transcriptional network (schema as exported from Metacore version 6.29). Color intensity indicates degree of regulation. Symbols refer to protein category classes according to Metacore version 6.29 classification (https://download.genego.com/files/A4_MetaCore_qrg_en.pdf). **(b)** Graph of qRT-PCR analysis of FASN, CD36, LPL, and FABP4 in nerves of P60 CT and cMU mice. Data normalized to β-actin and to the mean of CT. Data points represent *n* = 3 mice for CT and *n* = 4 mice for cMU (unpaired two-tailed two sample Student’s *t* test, FASN, P = 0.0203, *t* = 3.350; CD36, P = 0.0023, *t* = 5.711; LPL, P = 0.0123, *t* = 3.823; FABP4, P = 0.0161, *t* = 3.567); *, P < 0.05; **, P < 0.01. **(c)** Immunoblotting and corresponding graph showing decreased expression of FABP4 in lysates of sciatic nerves from P40 cMU compared with CT mice. Immunoblots normalized to β-actin. *n* = 3 blots, each data point representing an independent experiment with an independent set of lysates from three CT and three cMU mice (unpaired two-tailed two sample Student’s *t* test, P = 0.0045, *t* = 5.764); **, P < 0.01. Bars represent mean ± SEM.

De novo synthesis of FAs may induce myelination through various mechanisms, such as contributing to lipid availability, by itself, regulating signaling pathways, or directly regulating activity of transcription factors. Indeed, FAs bind to and induce a conformational change that leads to recruitment of cofactors and modulation of activity of the nuclear PPARs (PPARα, β, and γ; Ahmadian et al., 2013). We reasoned that decreased availability of FAs and derived lipids in the PNS of FASN mutant mice could translate into reduced transcriptional activity of PPARs. We tested this hypothesis through a transcriptome Affymetrix screening, comparing gene expression in sciatic nerves of P60 mutants with those of control mice. Of the identified 276 regulated (at least 1.5-fold) transcripts, the vast majority (246) was down-regulated (complete list in Table S3). MetaCore (Thomson Reuters) analysis revealed prominent down-regulation of the PPARγ transcriptional network (Fig. 7 a). We confirmed these results by quantitative RT-PCR (qRT-PCR) analysis of selected prototypical PPARγ targets (fatty acid translocase [CD36], lipoprotein lipase [LPL], and fatty acid binding protein 4 [FABP4]; Everett et al., 2000; Ahmadian et al., 2013) using epineurium-stripped P60 sciatic nerves (Fig. 7 b). FABP4 protein levels were also reduced in lysates of sciatic nerves dissected from P40 mutants compared with controls by immunoblotting (Fig. 7 c). Based on these data, we propose that de novo FA synthesis in SCs activates the PPARγ transcriptional network.

### The PPARγ agonist rosiglitazone can partially rescue SC myelination in the setting of deficient endogenous FA synthesis

**Figure 8.**
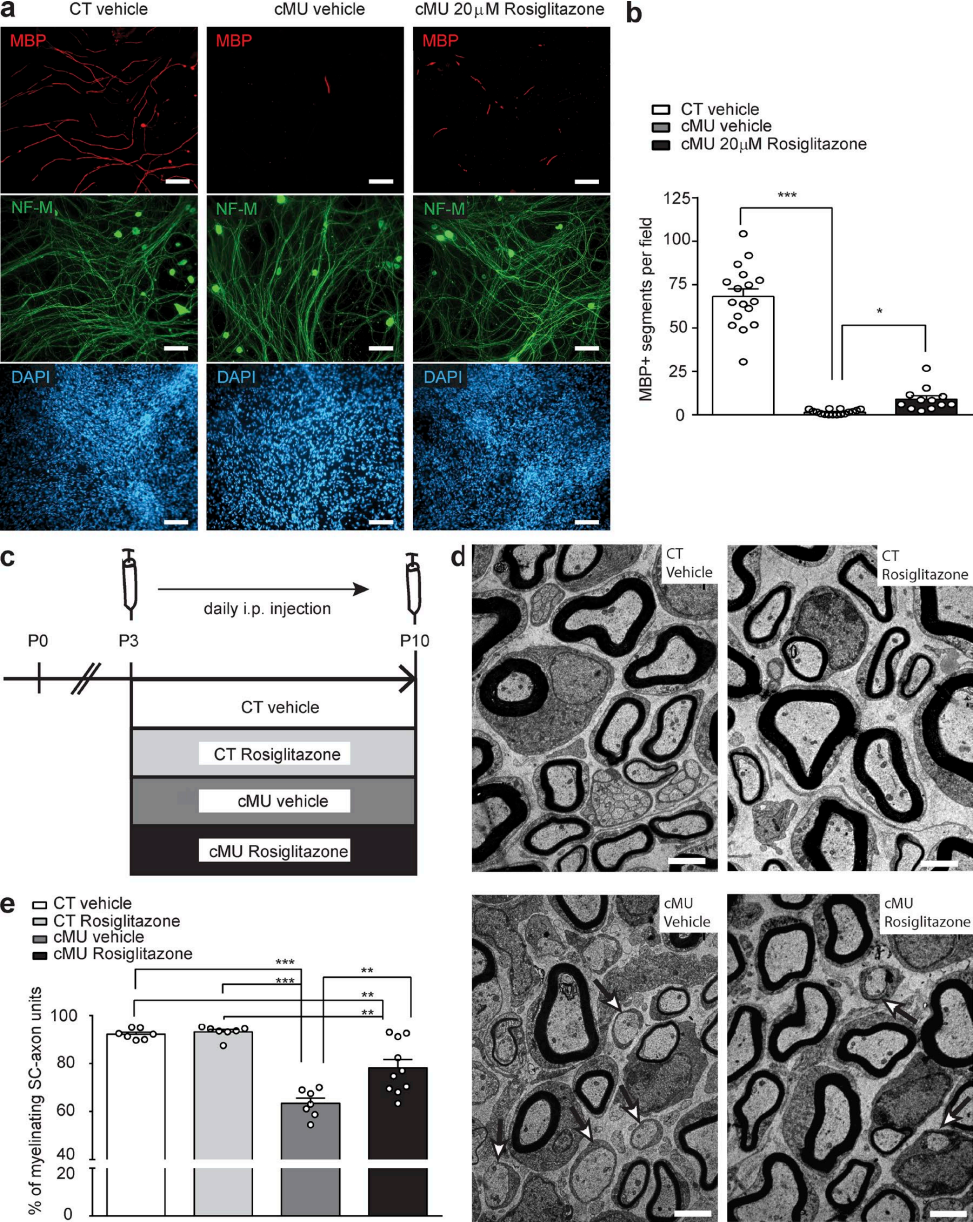
**Rosiglitazone administration partially rescues dysmyelination of FASN mutant SCs. (a)** Exemplary immunofluorescence of myelinating dissociated DR ganglia. Vehicle-treated control (CT), vehicle-treated mutant (cMU), and rosiglitazone-treated (20 µM) cMU mice. Treatment was started on the day when ascorbic acid was added. Bars, 100 µm. **(b)** Quantification of MBP-positive segments, 9 d after induction of myelination in CT and cMU treated cultures. Quantification was performed on four or more randomly selected fields of view per coverslip, on three to eight coverslips for each condition per experiment, in three independent experiments. Data points represent *n* = 17 coverslips of cultures from CTs treated with vehicle, *n* = 16 coverslips of cultures from cMUs treated with vehicle, and *n* = 12 coverslips of cultures from cMUs treated with rosiglitazone (Kruskal-Wallis, P < 0.0001, *H* = 37.78, with Dunn’s multiple comparison test; cMU vehicle vs. CT vehicle P < 0.0001; cMU rosiglitazone vs. cMU vehicle P = 0.0289); ***, P < 0.001; *, P < 0.05. **(c)** Schema depicting the experimental design of in vivo rosiglitazone or vehicle injections. **(d)** Exemplary electron micrographs of sciatic nerve cross sections of vehicle- or rosiglitazone-injected CT and cMU mice. Examples of promyelinating SC-axon units indicated by arrows. Bars, 2 µm. **(e)** Corresponding graph depicting complete quantification on fully reconstructed, EM-recorded cross sections of the percentage of myelinated axons (among sorted SC-axon units). Data points represent *n* = 7 mice each, for vehicle-injected CT, rosiglitazone-injected CT, and vehicle-injected cMU mice, and *n* = 10 mice for rosiglitazone-injected cMU mice (one-way ANOVA, treatment P < 0.0001, *F_3,27_* = 27.65, with Tukey’s multiple comparisons test; cMU vehicle vs. CT vehicle P < 0.0001; cMU rosiglitazone vs. CT vehicle P = 0.0023; CT rosiglitazone vs. CT vehicle P = 0.9946; cMU vehicle vs. CT rosiglitazone P < 0.0001; cMU rosiglitazone vs. cMU vehicle P = 0.0012; cMU rosiglitazone vs. CT rosiglitazone P = 0.0011); **, P < 0.01; ***, P < 0.001. Bars represent mean ± SEM.

Our data show that de novo FA synthesis in SCs is required to build up a correct lipid pool in nerves and suggest that this effect is critical for the activity of the transcription factor PPARγ. PPARs, in particular PPARγ, have been implicated in SC differentiation and/or myelination (Yamagishi et al., 2008; Cao et al., 2012). Thus, we next addressed whether restoring PPARγ activity in SCs lacking FA synthesis would reverse the observed block in the onset of myelination. For this purpose, we first analyzed dissociated DR ganglion-based myelinating cultures obtained from FASN mutant and control mice (Fig. 8 a). Cultures were treated with rosiglitazone, a potent and selective PPARγ agonist (Murphy and Holder, 2000; Marciano et al., 2014), or with vehicle alone as control (Fig. 8 a). Cultures derived from mutant mice displayed a strong reduction in myelination compared with controls (Fig. 8, a and b). This effect was partially rescued upon treatment with 20 µM rosiglitazone from the time of myelination induction (Fig. 8, a and b). Based on these findings, we asked whether this outcome could be translated to the in vivo condition. Thus, we administered rosiglitazone or vehicle intraperitoneally to FASN mutant mice at the time of myelination onset (P3), daily for 7 d, and compared the treated mutants with control mice injected with rosiglitazone or vehicle (Fig. 8 c). Consistent with our previous experiments, vehicle-injected mutant mice displayed a reduced percentage of myelinating SC-axon units compared with vehicle-injected control mice (63.44 ± 2.17% in vehicle-treated mutants vs. 92.29 ± 0.81% in vehicle-treated controls; Fig. 8, d and e). Control mice injected with rosiglitazone showed no effect on myelination onset (93.22 ± 0.99% of SC-axon units at myelinating stage; Fig. 8, d and e). Notably, we observed a significant increase in the percentage of myelinating SC-axon units in rosiglitazone-treated compared with vehicle-treated mutant mice (78.26 ± 3.48% in rosiglitazone-treated mutants vs. 63.44 ± 2.17% in vehicle-treated mutants; Fig. 8, d and e). To address the effect of rosiglitazone on selected prototypical PPARγ targets, we analyzed a set of treated mice by qRT-PCR. We found significantly increased expression of FABP4 (1.40 ± 0.30 in Rosiglitazone-treated mutants vs. 0.19 ± 0.17 in vehicle-treated mutants; Fig. S4 a). Other analyzed targets showed more variable expression not reaching significance (CD36: 0.60 ± 0.16 in rosiglitazone-treated mutants vs. 0.25 ± 0.11 in vehicle-treated mutants; LPL: 0.30 ± 0.08 in rosiglitazone-treated mutants vs. 0.21 ± 0.06 in vehicle-treated mutants; Fig. S4 a). We conclude that treatment with the PPARγ agonist rosiglitazone was able to partially release the block in myelination onset caused by the lack of FASN expression in SCs.

## Discussion

Myelination is metabolically a highly demanding process, requiring glial cells to timely and precisely increase and coordinate RNA, protein and lipid synthesis, protein targeting, and massive membrane production (Taveggia et al., 2010; Pereira et al., 2012; Nave and Werner, 2014; Monk et al., 2015; Herbert and Monk, 2017). Many aspects of the network of metabolic changes critical for successful myelination remain to be elucidated. In particular, how myelinating glial cells adapt to changes in lipid demands, i.e., the relative functional role of increased de novo lipogenesis versus lipid uptake, has not been fully addressed (Schmitt et al., 2015). In this study, we found that SCs do not substantially rely on FA synthesis for postnatal survival or proliferation. However, conditional depletion of FASN in SCs during embryonic development results in defective onset of myelination, impaired nerve lipid composition, and a chronic incapacity of SCs to myelinate efficiently.

The onset of PNS myelination is driven by the integration of multiple signaling axes, ultimately modulating the activity of master gene regulators of myelination (Jessen and Mirsky, 2005; Svaren and Meijer, 2008; Pereira et al., 2012; Glenn and Talbot, 2013). FAs and derived lipids also modulate transcription by binding to PPARs, inducing a conformational change that leads to recruitment of cofactors and modulation of their activity (Ahmadian et al., 2013). The interplay of FA synthesis and PPAR activation has been intensively studied in adipocytes, macrophages, and liver cells (Lodhi et al., 2012; Ahmadian et al., 2013), but a role for PPARγ has been suggested also in oligodendrocyte progenitor proliferation and differentiation by in vitro studies (Paintlia et al., 2010; Bernardo et al., 2013). PPARγ is expressed by SCs (Cao et al., 2012), so we reasoned that decreased availability of endogenous FAs and their downstream lipids in the PNS of FASN mutant mice may ultimately translate into reduced PPARγ transcriptional activity similar to what was described for adipocytes (Lodhi et al., 2012). Our results support this hypothesis. Moreover, we provide evidence that FASN-dependent lipogenic activation of the PPARγ transcriptional program is indeed likely to be important for the onset of SC myelination, because rosiglitazone administration was sufficient to ameliorate the dysmyelinating phenotype observed in FASN mutant SCs ex vivo and in vivo. We note that off-target interactions of rosiglitazone have been reported (Hoffmann et al., 2012), and we cannot currently exclude that such effects may play a role in our experimental settings. Our data indicate that the accurate availability of FAs is not only required for correct myelin membrane production, but FA synthesis in SCs is of broader significance. We anticipate that de novo synthesized FAs may also contribute to PNS myelination by modulating additional pathways, e.g., targeting proteins to membranes by palmitoylation. Addressing other contributing mechanisms in FA-mediated regulation of myelination will be pursued in future investigations.

The mTORC1/SCAP/SREBP axis has been inferred to be the main regulator of FASN transcription in myelinating SCs, and accordingly, SCAP and Raptor SC-conditional mutant mice showed early down-regulation of FASN expression (Verheijen et al., 2009; Norrmén et al., 2014). Nonetheless, neither mTOR, Raptor, SCAP, nor SREBP1c mutant mice displayed chronic impairments in myelination onset in a large subset of myelination-competent axons, resembling the findings we report here caused by a lack of FASN expression in SCs (Verheijen et al., 2009; Sherman et al., 2012; Norrmén et al., 2014; Cermenati et al., 2015). Rather, like many other previously studied lipogenic enzymes (Schmitt et al., 2015), the aforementioned proteins appear to be mainly critical regulators of myelin growth (Verheijen et al., 2009; Sherman et al., 2012; Norrmén et al., 2014; Schmitt et al., 2015). Currently unknown compensatory mechanisms might be at work in those mutants, leading to a delay rather than a block in myelination. Future work aimed at identifying the underlying molecular pathways is likely to be fruitful in dissecting the precise role of lipids and their metabolism in peripheral nerve myelination.

Unexpectedly, we found that dependence of SCs on endogenous de novo FA synthesis varies by developmental origin and/or location. SCs located in nerve roots (mostly derived from boundary cap cells [Maro et al., 2004; Aquino et al., 2006]) are more strongly reliant on SC-intrinsic FASN activity than SCs present in distal nerves (directly derived from the neural crest [Maro et al., 2004; Aquino et al., 2006]). Of particular interest in this context is our peculiar observation, specifically in FASN mutant sciatic nerves, that their associated epineurial adipocytes contained small and multiple lipid vesicles, a sign of ongoing lipolysis and lipid release. Moreover, we found that nerve roots lacked epineurial adipocytes. Thus, it is conceivable that in the absence of endogenous FA synthesis in distal nerve SCs, increased lipid support from epineurial adipocytes has a compensatory role to promote myelination. These findings highlight various compensatory mechanisms used by glial cells defective in lipid synthesis. In the CNS, oligodendrocytes with a shortage in lipid synthesis appear to be supported by horizontal lipid flux from astrocytes (Camargo et al., 2017). Failure of astrocytes to provide this support results in increased uptake of dietary lipids by myelinating oligodendrocytes (Camargo et al., 2017). Regarding SCs, our results do not exclude the possibility that in distal nerves and nerve roots different subpopulations with diverse metabolic demands may exist. These issues warrant further investigation because they are not only relevant to our basic understanding of the mechanisms of peripheral nerve myelination in development but they may also have an impact on physiological regulations related to lipid metabolism under different conditions in peripheral nerves, its diseases, and after injury.

Lipogenesis in myelinating glial cells has been thought to be mainly relevant for proper myelin growth and long-term axon-myelin maintenance (Schmitt et al., 2015). Our data reveal that a lipogenic switch in developing SCs is essential for correct initiation and efficiency of myelination. Overall these findings provide fundamentally novel information about the regulation of myelination, establishing the basis for future studies into the complex lipogenic regulation of glial function and for gaining further insights into the pathophysiology of lipid-associated diseases.

## Materials and methods

### Resource sharing

The *Fasn* floxed mice are available from the Semenkovich laboratory after executing a Material Transfer Agreement with Washington University. The *Dhh*-Cre mice are available from Jackson Labs (stock no. 012929).

### Animal models

Mice (*Mus musculus*) homozygous for the FASN (*Fasn*) floxed allele (Chakravarthy et al., 2005; strain of origin: 129X1/SvJ, subsequently crossed with 129X1/SvJ*C57BL/6*DBA) were crossed with mice expressing Cre recombinase under the control of the *Dhh* promoter (Jaegle et al., 2003), to obtain *Cre*^+^ *Fasn^lox/lox^* mice (hereafter called mutant mice), and *Cre*^−^ *Fasn^lox/lox^* or *Fasn^lox/wt^* (hereafter called control mice). *Fasn* floxed mice were backcrossed for at least three generations and *DhhCre* mice for more than 10 generations with C57BL/6 mice. Both male and female mice were used throughout all experiments. Mice were group-caged and kept in a 12-h light/dark cycle. Littermates and age-matched mice were assigned to experimental groups according to age and genotype. Animals were fed either an STD (3437; KLIBA NAFAG; Provimi KLIBA) or an HFD containing 60% fat calories (2127; KLIBA NAFAG, Provimi KLIBA), as previously described (Camargo et al., 2012). Pregnant mice received an HFD from day 14 of gestation, during pregnancy and lactation, until pups were weaned 3 wk after birth. At weaning, pups were separated and housed by gender, while continuously receiving the HFD. Mice fed an HFD were sacrificed at 6 wk of age. FAs and cholesterol content (expressed as percentage values) in the administered HFD and STD are listed in Table S1.

All genotypes were determined by PCR on genomic DNA (Fasn primers: 5′-GGATAGCTGTGTAGTGTAACCAT-3′ and 5′-GGTCACCCAGCAGGAAAGGGC-3′; Cre primers: 5′-TTCCCGCAGAACCTGAAGATGTTCG-3′ and 5′-GGGTGTTATAAGCAATCCCCAGAAATG-3′).

All animal experiments were performed with the approval and in strict accordance with the guidelines of the Zurich Cantonal Veterinary Office.

### In vivo rosiglitazone injections

Mice received daily intraperitoneal injections over 7 d (P3 to P9) of rosiglitazone (25 mg/kg/day at P3 and P4, and 50 mg/kg/day from P5 to P9; 71742; Cayman Chemicals) or vehicle (1× PBS) alone as control. Injected mice were sacrificed 12 h (at P10) after the last injection and tissues dissected for subsequent analysis.

### Behavior analysis

P40 control (*n* = 9) and mutant (*n* = 9) mice were tested during the light cycle. For motor behavior, a Rotarod (Ugo Basile) was set to accelerate from 4 to 40 rpm over 5 min. Four separate trials were scored at intervals of 1 h. The average latency to fall from the rotating rod over three repeats run in succession was calculated for each trial. Subsequently, the same set of mice was used to test neuromuscular function with a Grip-Strength Meter (Ugo Basile). Mice were allowed to grip the metal bar with forelimbs and gently pulled, and the mean peak strength over five trials run in succession was calculated.

### Myelinating dissociated DR ganglia cultures

Myelinating dissociated DR ganglia were established on Matrigel (BD Biosciences)-coated coverslips as previously described (Trimarco et al., 2014). For each experiment, DR ganglia were dissected from E13.5 control and mutant mice into L15 (Life Technologies) medium and subsequently digested in 0.25% Trypsin/EDTA (Life Technologies) for 45 min. Cells from mice of the same genotype were pelleted together and resuspended in Neurobasal (Life Technologies) supplemented with 10% B27 (Life Technologies), 5 g/liter d-glucose (Sigma-Aldrich), 50 ng/ml nerve growth factor (mNGF 2.5S; Millipore), 2 mM l-glutamine (Life Technologies), and penicillin/streptomycin (Life Technologies). Cells equivalent to 1.5 dissociated DR ganglia were plated per coverslip, and resuspension medium was changed every second day. Myelination was induced after 9 d in vitro by adding 50 µg/ml ascorbic acid (Sigma-Aldrich) to MEM/Glutamax (Life Technologies) medium, supplemented with 10% FBS (Life Technologies), 5 g/liter d-glucose (Sigma-Aldrich), and 50 ng/ml 2.5S NGF (Millipore) in the absence of antibiotics. Cells were treated with either 20 µM rosiglitazone (71742; Cayman Chemicals) or an equal volume of vehicle (1× PBS). The medium with the treatment was replaced every second day, and coverslips were processed after 18 d in vitro for immunofluorescent detection of MBP, NF-M, and DAPI.

### Antibodies

The following antibodies were used: mouse anti–β-actin (WB 1:2,000, clone AC74; A2228; Sigma-Aldrich), goat anti–contactin 1 (IHC 1:40; AF904; R&D Systems), rabbit anti–FABP4 (WB 1:1,000, clone D25B3; 3544; Cell Signaling), rabbit anti–FASN (WB 1:1,000, IHC 1:200; Ab22759; Abcam), mouse anti–Kv1.2 (IHC 1:200; Ab192758; Abcam), rat anti–MBP (ICC and IHC 1:200, clone 12; MCA409S; Serotec), rabbit anti–neurofascin186 (IHC 1:300; Ab31719; Abcam), mouse anti–neurofilament M (ICC and IHC 1:200, clone NN18; MAB5245; Millipore), rabbit anti–oct6 (WB 1:1,000, provided by D. Meijer, University of Edinburgh, Edinburgh, Scotland, UK; Zwart et al., 1996), chicken anti-P0 (IHC 1:250; AB9352; Millipore), rabbit anti-PMP22 (WB 1:2,000, in-house; Snipes et al., 1992), goat anti–mouse IgG HRP coupled (WB 1:5,000; 115.035.003; Jackson Laboratories), goat anti–rabbit IgG HRP coupled (WB 1:5,000; 111.035.003; Jackson Laboratories), Isolectin B4 from *Griffonia simplicifolia*–conjugated to Alexa Fluor 594 (IHC 1:100; I21413; Invitrogen), donkey anti–goat IgG Alexa Fluor 488 coupled (IHC 1:500; A11055; Life Technologies), donkey anti–mouse IgG Alexa Fluor 546 coupled (IHC 1:500; A10036; Life Technologies), donkey anti–rabbit IgG Alexa Fluor 647 coupled (IHC 1:500; A31573; Life Technologies), goat anti–chicken IgG H-L Alexa Fluor 488 coupled (IHC 1:300; 103.545.155; Jackson Laboratories), goat anti–mouse IgG H-L Alexa Fluor 488 coupled (IHC 1:300; 111.545.144; Jackson Laboratories), goat anti–rabbit IgG H-L Cy3 coupled (IHC 1:300; 111.165.144; Jackson Laboratories), goat anti–Rat IgG H-L Cy5 coupled (IHC 1:300; 112–175-167; Jackson Laboratories), and goat anti–rat IgG H-L Alexa Fluor 594 coupled (IHC 1:300; 112–585-143; Jackson Laboratories).

### Immunoblotting

Tissues were homogenized with chilled mortar and pestle in CHAPS lysis buffer (50 mM NaH_2_PO, pH 8.0, 150 mM NaCl, 0.5% CHAPS, 0.2% NaF, 0.01% Na_3_VO_4_, 0.04% EDTA, and proteases inhibitor cocktail [Roche]). Protein concentration was determined by NanoDrop Lite (NanoDrop Technologies). 10 µg of protein per lane was resolved on precasted NuPAGE 4–12% gradient gels or on linear SDS-PAGE and transferred to polyvinylidene difluoride membranes. Membranes were processed using standard immunoblotting procedures. Proteins were detected by binding primary antibodies to HRP-conjugated secondary antibodies and subsequently developed with Supersignal West Pico Chemiluminescent Substrate (Pierce). Chemiluminescent signal was detected, and images were acquired onto a Fusion FX7 system equipped with FusionCapt Advance FX7 software (Vilber Lourmat). Only images containing bands with no saturated pixels, confirmed by the software detection system, were used for subsequent quantification. Densitometry and quantification of relative levels were performed with ImageJ software. Full-length blots are shown in Fig. S4 b. Size markers refer to All Blue Precision Protein Standards (Biorad).

### Transmission and scanning EM

Mice were anaesthetized by a terminal intraperitoneal injection of Eskonarkon (pentobarbital, 10% in saline solution [0.9% NaCl]), and perfused with 0.1 M phosphate buffer, pH 7.4, followed by 2.5% glutaraldehyde and 4% paraformaldehyde in 0.1 M phosphate buffer. Fixed tissues were dehydrated through a graded acetone series, postfixed in 2% osmium tetroxide overnight, and embedded in Spurrs resin (Electron Microscopy Sciences). Semithin sections were stained with 1% toluidine blue (Sigma-Aldrich) for light microscopy analysis. Ultrathin sections (65 nm for transmission electron microscopy [TEM] and 99 nm for scanning EM) were cut on a UC-7 (Leica) or a Reichert-Jung Ultra cut E ultramicrotome. Sections were deposited onto copper grids with carbon film (Electron Microscopy Sciences) for TEM or onto indium tin oxide coverslips (Optic Balzers) for scanning EM, and counterstained with 2% uranyl acetate and 1% lead citrate. For TEM, multiple random fields of sciatic nerves/roots were imaged with a Morgagni (FEI) transmission electron microscope. For scanning EM, the entire surface of sciatic nerve/root cross sections was imaged using the in-lens detector of a Zeiss Merlin FEG scanning EM operating at 2 KeV, attached to the ATLAS module (Zeiss). The entire nerve/root panorama was acquired as multiple individual images, with overlap between adjacent ones. Alignment and merging of the images were performed using FIJI (ImageJ) and Photoshop CS5 or CS6 (Adobe). Brightness and contrast of the images were adjusted for optimal detection of the structures.

### Immunofluorescence, TUNEL, and EdU-proliferation assay

For immunohistochemistry, mice were anaesthetized by terminal intraperitoneal injection of Eskonarkon (pentobarbital, 10% in saline solution) and intracardially perfused with 1× PBS followed by 4% paraformaldehyde and 5% sucrose in 1× PBS. Dissected tissues were postfixed in 4% paraformaldehyde and 5% sucrose in 1× PBS overnight and cryoprotected in 30% sucrose in 1× PBS for 24 h. Tissues were embedded in Tissue-Tek OCT (Sakura). 10-µm sections were cut on a cryostat and transferred to SuperFrost Plus (Thermo Scientific)–coated slides.

All sections from samples to be compared were processed simultaneously. Sections were blocked for 1 h with 10% donkey (for node of Ranvier stainings) or goat serum (Invitrogen) and 1% Triton X-100, in 1× PBS, and incubated with primary antibodies overnight (4°C). Tissue sections were washed (1× PBS) and incubated with secondary antibodies (1 h at room temperature). After incubation with DAPI and washing (1× PBS), sections were coverslipped with Shandon ImmuMount (Thermo Scientific). Cell proliferation was analyzed using the Click-iT EdU assay (Invitrogen), according to the manufacturer’s protocol. Apoptotic cell death was analyzed by TUNEL staining using biotin-labeled UTP and Alexa Flour 546–conjugated streptavidin complex according to the manufacturer’s instructions (Roche Diagnostics). Cell proliferation and apoptosis were quantified with ImageJ software. The experimenter was blind to the genotype.

### Oil Red O staining

Dissected tissues were processed as previously described (Nadra et al., 2012). In brief, they were washed in 1× PBS and incubated 10 min in Oil Red O (O1391; Sigma-Aldrich; 0.5% in isopropanol), previously diluted 3:2 in water, and filtered. After washing 10 min in water, tissue was mounted on Superfrost Plus glass slides (Thermo Scientific) with Vectashield Mounting medium (Vectashield) for subsequent imaging under light microscopy.

### Microscope image acquisition

Images of tissue sections stained by immunofluorescence, Click-EdU labeling, and TUNEL staining were acquired by using the Zen 2 software (Zeiss) on an AxioImager M2 microscope (Zeiss) equipped with a high-resolution camera (pco.edge; sCMOs Cameras) and with either a Plan Apochromat 20×/0.8 air objective (420651-9911; Zeiss) or with a 40× Plan Apochromat 40×/0.95 air objective (420661-9970; Zeiss). Single-channel images were merged to create composite multichannel images with ImageJ software.

Images of Oil Red O– and toluidine-stained tissue sections were acquired by using AxioVision 4.8 software (Zeiss) on a Zeiss Axioskop 2 microscope equipped with an AxioCam HRc camera (Zeiss) and with either a Plan-Neofluar 20×/0.5 air objective (440341-9904; Zeiss), a Plan-Neofluar 40×/1.30 oil immersion objective (1022-818; Zeiss), or a Plan-Neofluar 100×/1.30 oil immersion objective (440480; Zeiss).

Images of nodal stainings were acquired on an LSM 780-FCS laser scanning confocal microscope (Zeiss) equipped with a Plan-Apochromat DIC 63×/1.4 oil immersion objective (421782-9900-799; Zeiss) using an argon laser (488 nm), a solid-state laser (561 nm), and a helium-neon laser (633 nm). For signal detection two Quasar photomultipliers and 32 photomultiplier GaAsP detectors were used.

All images were acquired at room temperature (20–24°C).

### Lipidomics

Chemicals were purchased either from Merck or Sigma-Aldrich. Lipid standards were obtained from Avanti Polar Lipids. Analysis of lipid species was performed according to published methods (Fauland et al., 2011). Samples were extracted with methyl tert-butyl ether (Matyash et al., 2008). Lipid extracts were evaporated and resuspended in 1 ml chloroform/methanol (1:1, vol/vol). Each lipid extract was then split for analysis of total FAs (350 µl), positive electrospray ionization (ESI) liquid chromatography-tandem mass spectrometry (LC-MS/MS; 18 µl) and negative ESI LC-MS/MS (18 µl). Lipid extracts for LC-MS/MS analysis were evaporated and spiked with a mix of quantitative LIPID MAPS internal standards, and 5-µl spiked samples were injected onto a 1.9-µm Hypersil GOLD C18 (Thermo Scientific), 100 × 1 mm HPLC column mounted in an Accela HPLC instrument (Thermo Scientific). Data acquisition was performed by Fourier transform ion cyclotron resonance–MS (LTQ-FT model; Thermo Scientific) full scans at a resolution of 200 k and <2 ppm mass accuracy with external calibration. Nominal mass resolution product ion spectra were acquired in preview mode at the LTQ. A chromatography with electron impact mass spectrometry of total FAs (free plus esterified) was performed. Lipid extracts were dried and suspended in 1 ml methanolic NaOH. After 10-min incubation at 80°C, samples were cooled for 5 min on ice. Then, 1 ml BF3 was added and incubated for 10 min at 80°C. FA methyl esters were extracted with 1 ml saturated NaCl and 2 ml hexane. The hexane phase was dried, and methyl esters were dissolved in 1.5 ml hexane. A Trace–dual-stage quadrupole GC-MS (Thermo Scientific) equipped with a 30-m column (model TR-FAME; Thermo Scientific) was used for analysis. Helium was used as the carrier gas at a flow of 1.3 ml/min, in split mode, at 250°C injector temperature. An initial oven temperature of 150°C was held for 0.5 min, and then the temperature was increased to 180°C at a rate of 10°C/min. This was followed by a further increase to 190°C at a rate of 0.5°C/min and then increased to 250°C at a rate of 40°C/min and kept for 3 min. The mass spectrometer was run in electron impact mode, and FAs were detected in full scan of 80–400 mass per charge number. Source temperature was set to 250°C and the transfer line temperature to 200°C. Internal standards used in LC-MS were (LIPID MAPS identification number [LM], lipid shorthand nomenclature [PE, phosphatidylethanolamine; PS, phosphatidylserine; PC, phosphatidylcholine; PI, phosphatidylinositol], amount/sample): LM-1100, PE 12:0/13:0, 160 pmol; LM-1102, PE 17:0/20:4, 160 pmol; LM-1103, PE 21:0/22:6, 160 pmol; LM-1104, PE 17:0/14:1, 160 pmol; LM-1302, PS 17:0/20:4, 240 pmol; LM-1300, PS 12:0/13:0, 240 pmol; LM-1304, PS 17:0/14:1, 240 pmol; LM-1000, PC 12:0/13:0, 200 pmol; LM-1002, PC 17:0/20:4, 200 pmol; LM-1003, PC 21:0/22:6, 200 pmol; LM-1004, PC 17:0/14:1, 200 pmol; LM-1601, LPC 17:1, 80 pmol; LM-6002, sphingolipid mix, 120 pmol; LM-1500, PI 12:0/13:0, 320 pmol; LM-1502, PI 17:0/20:4, 320 pmol, LM-1504, PI 17:0/14:1, 320 pmol.

Data were processed by Lipid Data Analyzer software as previously described (Hartler et al., 2011), relying on exact mass and retention time. Annotation of lipid species followed the shorthand nomenclature of the International Lipid Classification and Nomenclature Committee (Liebisch et al., 2013). During the lipid extraction procedure, the fraction containing proteins was lyophilized. Proteins were dissolved in lysis buffer (1% Rapigest [Waters/Millipore], Tris, pH 7.4, and 0.1 mM EDTA) and quantified onto a NanoDrop Lite (NanoDrop Technologies) spectrophotometer. Lipid amounts were normalized to the sample total protein content.

### RNA isolation

Dissected tissue was grinded in Trizol (Sigma-Aldrich) into a liquid-nitrogen precooled mortar. Grinded powder was resuspended in Trizol and RNA extracted by chloroform/isopropanol standard purification protocol. Quantity and quality of isolated RNA were determined with NanoDrop ND 1000 (NanoDrop Technologies), Qubit 2.0 Fluorometer (Life Technologies), and Bioanalyzer 2100 (Agilent). Further processed samples had a 260:280-nm ratio within 1.8–2.1 and 28S:18S ratio within 1.5–2.

### Affymetrix transcriptome analysis

Amplified RNA (aRNA) was prepared with a GeneAtlasTM 3′IVT Express kit (Affymetrix) according to the manufacturer’s instructions. RNA samples (100 ng) were reverse-transcribed into first-strand cDNA with T7 oligo(dT) primer. DNA polymerase and RNaseH were used simultaneously to synthesize double-stranded cDNA and degrade the RNA. Double-stranded cDNA was amplified in vitro and labeled with biotin to obtain biotin-modified aRNA, which was further purified by using RNA Binding Beads to remove unincorporated nucleoside triphosphates, salts, enzymes, and inorganic phosphate. aRNA yield and size distribution were determined with NanoDrop ND 1000 and Bioanalyzer 2100. 10 µg of biotin-labeled aRNA was fragmented to reach a range of 35–200-nt aRNA fragments. For array hybridization, biotin-labeled aRNA samples were mixed in 150 µl WT Hybridization Cocktail (Affymetrix) containing Hybridization Controls and Control Oligonucleotide B2 (Affymetrix). Samples were hybridized to Affymetrix MG-430 PM Array Strip (Affymetrix) in a GeneAtlas Hybridization Station (Affymetrix) for 16 h at 45°C. Arrays were washed in a GeneAtlas Fluidics Station (Affymetrix). The fluorescent intensity emitted by the labeled targets was measured onto a GeneAtlas Imaging Station (Affymetrix). Raw data were exported to Excel and subsequently analyzed by GeneSpring GX (Agilent) by applying an RMA summarization algorithm with the baseline set to the median of all samples. Entities were filtered based on their corrected p-values calculated asymptotically with ANOVA analysis and Benjamini-Hochberg false discovery rate for multiple testing correction. The p-value cut-off was set at 0.05. A final threshold of ≥1.5-fold increase or decrease in the expression level of each single transcript was applied. Pathway analysis was run using Metacore (Thompson Reuters) software.

Microarray data are MIAME compliant and have been deposited at the GEO repository database with accession code GSE71373.

### Quantitative real-time PCR

RNA samples were reverse-transcribed to cDNA with oligo(dT) random primers in the presence of RNaseOUT (Invitrogen) with Superscript III RT (Invitrogen) or with the Maxima RT kit (Thermo Scientific). Targeted sequences were amplified with exon/exon boundaries spanning probes and detected by measurement of SYBRgreen on a LightCycler 480 (Roche). Cp values were determined with the LightCycler 480 software (Roche). Sequences of used primers are available in Table S2.

### Statistical analysis

Statistics were analyzed by using GraphPad Prism v6.01. Gaussian distribution of data was tested with a D’Agostino-Pearson omnibus normality test. When Gaussian distribution could not be tested, because of a low number, data were assumed to be normally distributed. Variance was assumed to be equal between groups of data. For normally distributed data, statistical significance was determined by using an unpaired two-sample Student’s *t* test for two-group comparisons. Multiple groups analysis was performed with one- or two-way ANOVA and post hoc test as detailed in text and figures. Data show mean ± SEM of three independently run experiments unless otherwise specified in text and figures. Significance was set at *, P < 0.05; **, P < 0.01; and ***, P < 0.001. No statistical methods were used to predetermine sample size, but our sample sizes are similar to those generally used in the field.

### Data and software availability

Microarray datasets have been deposited in the National Center for Biotechnology Information’s Gene Expression Omnibus database, accession no. GSE71373. Full-length Western blots are shown in Fig. S4 b. All software is freely or commercially available.

### Online supplemental material

Fig. S1 shows that de novo FA synthesis in SCs is dispensable toward SC early postnatal survival and proliferation and not essential for axonal maintenance. Fig. S2 shows that SCs in distal nerves, engaged either with predominantly sensory or motor fibers, appear equally dependent on de novo FA synthesis for efficient onset of myelination. Fig. S3 shows lipid profiles of sciatic nerves of control and FASN mutant mice. Fig. S4 shows expression of PPARγ targets after rosiglitazone treatment and full-length immunoblots. Table S1 shows FAs and cholesterol content (expressed as percentage values) in the administered HFD and STD. Table S2 shows qRT-PCR probes and primers. Table S3 shows regulated transcripts in sciatic nerves of P60 mutant mice compared with control mice identified by Affymetrix Transcriptome Analysis.

## Supplementary Material

Supplemental Materials (PDF)

Table S3 (Excel)
