# Supplementary material for: De novo fatty acid synthesis by Schwann cells is essential for peripheral nervous system myelination
Source: J Cell Biol. 2018 Apr 2;217(4):1353–68. doi: 10.1083/jcb.201706010 (PMC5881495; doi:10.1083/jcb.201706010)
Supplement: Supplemental Materials (PDF) [file JCB_201706010_sm.pdf]

Montani et al., https://doi.org/10.1083/jcb.201706010

JCB

JOURNAL OF CELL BIOLOGY

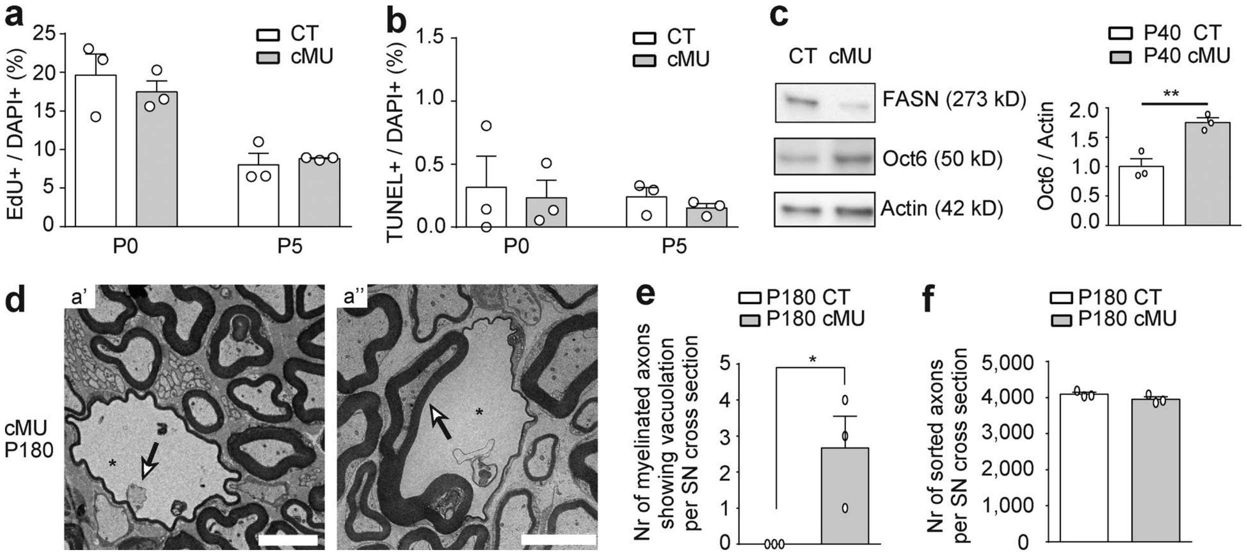

Figure S1. **De novo FA synthesis in SCs is dispensable toward SC early postnatal survival and proliferation and not essential for axonal maintenance.** **(a)** No detected statistically significant difference in the percentage of proliferating cells in cryosections of P0 and P5 sciatic nerves of conditional mutant (cMU) mice compared with control (CT) mice. Data points represent $n = 3$ mice for each, cMU and CT (unpaired two-tailed two sample Student's t test, P0 P = 0.5179, $t = 0.7083$; P5 P = 0.6171, $t = 0.5412$). **(b)** No statistically significant difference was detected in the percentage of apoptotic cells in cryosections of P0 and P5 sciatic nerves of cMU mice compared with CT mice. Data points represent $n = 3$ mice for each, cMU and CT (unpaired two-tailed two sample Student's t test, P0 P = 0.7850, $t = 0.2918$; P5 P = 0.3414, $t = 1.079$). **(c)** Increased expression levels of the transcription factor Oct6 in protein lysates from sciatic nerves of P40 cMU mice compared with CT mice. Immunoblots were normalized to β-actin. $n = 3$ blots, each data point represents an independent experiment with an independent set of lysates from three CT and three cMU mice (unpaired two-tailed two sample Student's t test, Oct6 P = 0.0083, $t = 4.855$); **, P < 0.01. **(d)** EM images depicting compressed myelinated axons (examples indicated by arrows) presumably because of vacuolization (examples marked by stars) in sciatic nerves of P180 mutant mice (cMU). Bar, 5 µm. **(e)** Graph with quantification of the number of myelinated axons with vacuolation per fully reconstructed sciatic nerve cross section, at P180. $n = 3$ mice for each, control (CT) and cMU (unpaired two-tailed two sample Student's t test, P = 0.0390, $t = 3.204$), *, P < 0.05. **(f)** Graph showing quantification of total number of sorted axons per fully reconstructed sciatic nerve cross section at EM level at P180. $n = 3$ mice for each, CT and cMU (unpaired two-tailed two sample Student's t test, P = 0.1652, $t = 1.696$). Bars represent mean ± SEM.

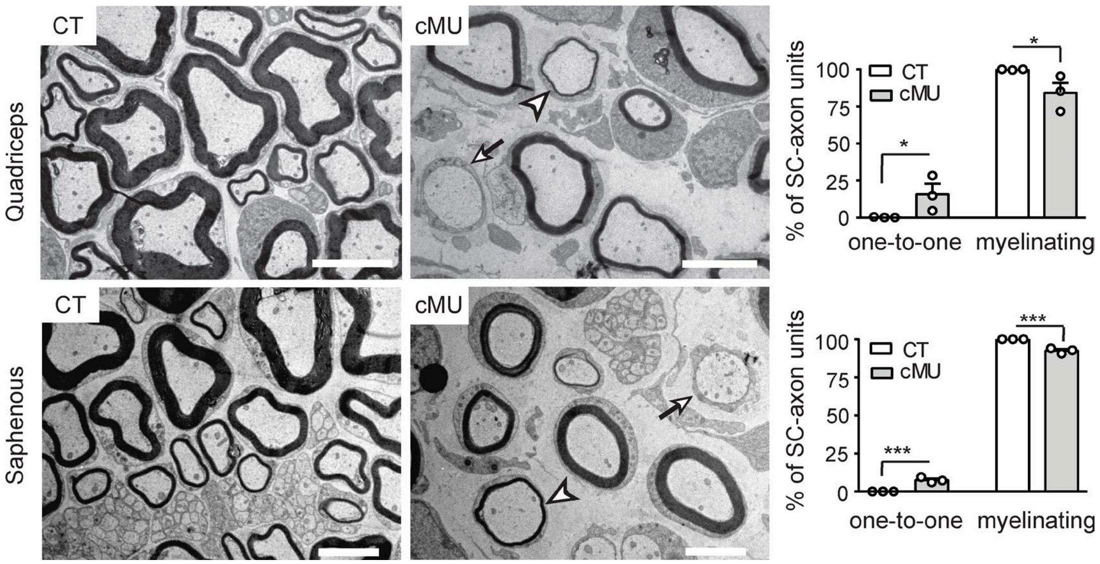

Figure S2. **SCs in distal nerves, engaged either with predominantly sensory or motor fibers, appear equally dependent on de novo FA synthesis for efficient onset of myelination.** Representative EM images of P24 mutant (cMU) and control (CT) saphenous and quadricep nerve cross sections. Whereas individual large-caliber axons (>1 μm in diameter) are myelinated in CT nerves, SC-axon units at the promyelinating stage are still present in cMU (examples indicated by arrows). Thinly myelinated axons are also present in cMU nerves (examples indicated by arrowheads). Bars, 5 μm. Corresponding graphs show quantification of the percentage of SC-axon units at the one-to-one versus myelinating stage. Significance was calculated compared with controls. Data points represent $n = 3$ mice for each, CT and cMU (unpaired one-tailed two sample Student's t test [hypothesis of up-regulation of one-to-one SC-axon units in cMU compared with CT, as previously observed in roots and sciatic nerves, see Figs. 2 and 3], quadricep $P = 0.0420$, $t = 2.288$; saphenous $P = 0.0008$, $t = 7.524$); ***, $P < 0.001$; *, $P < 0.05$. Bars represent mean ± SEM.

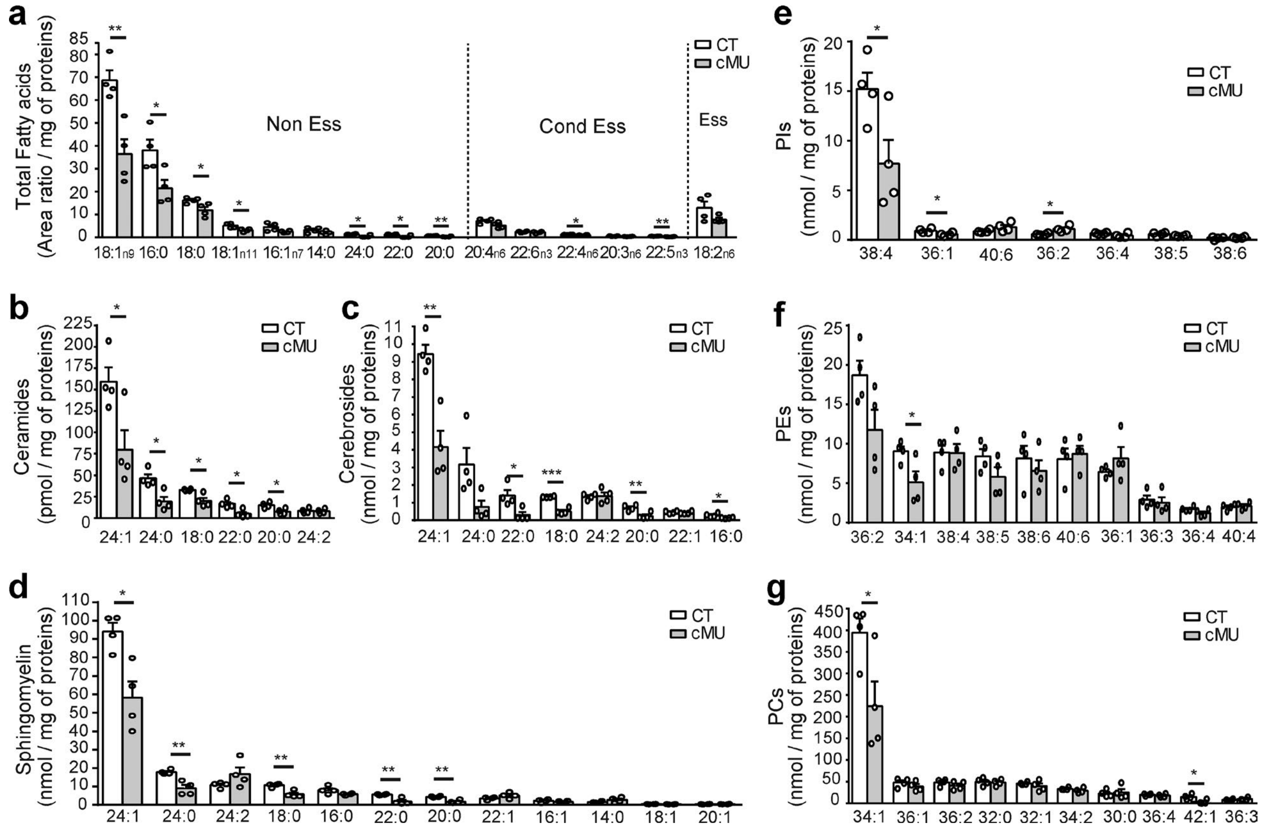

Figure S3. **Lipid profiles of sciatic nerves of control and FASN mutant mice.** Lipids extracted from sciatic nerves of P24 control (CT) and mutant (cMU) mice were identified and quantified by LC-MS. **(a)** Depicted are individual total FAs (TFA), subdivided in nonessential (Non Ess; which can be synthesized), essential (Ess; which must be taken up through the diet), and conditional essential (Cond Ess; which can either be taken up through the diet or synthesized from essential FAs). Of the two essential FAs, linoleic acid and α-linolenic acid, only linoleic acid (18:2n6) showed a quantity detectable by our analysis. Individual species of all identified ceramides (b), cerebrosides (c), sphingomyelin (d), phosphatidylinositols (PIs; e), and of the 10 highest phosphatidylethanolamines (PEs; f) and phosphatidylcholines (PCs; g) are shown. Lipid amounts were normalized to the total protein content of the sample. Significance was calculated compared with controls. Data points represent *n* = 4 mice for each, CT and cMU (unpaired two-tailed two sample Student's *t* test, TFAs—18:1n9 P = 0.0062, *t* = 4.126; 16:0 P = 0.0313, *t* = 2.796; 18:0 P = 0.0331, *t* = 2.754; 18:1n11 P = 0.0228, *t* = 3.042; 16:1n7 P = 0.0796, *t* = 2.108; 14:0 P = 0.2053, *t* = 1.420; 24:0 P = 0.0212, *t* = 3.096; 22:0 P = 0.0214, *t* = 3.091; 20:0 P = 0.0058, *t* = 4.182; 20:4n6 P = 0.0654, *t* = 2.251; 22:6n3 P = 0.5091, *t* = 0.7017; 22:4n6 P = 0.0339, *t* = 2.735; 20:3n6 P = 0.0867, *t* = 2.046; 22:5n3 P = 0.0064, *t* = 4.100; 18:2n6 P = 0.1292, *t* = 1.758; ceramides—24:1 P = 0.0305, *t* = 2.817; 24:0 P = 0.0111, *t* = 3.621; 18:0 P = 0.0141, *t* = 3.422; 22:0 P = 0.0124, *t* = 3.527; 20:0 P = 0.0160, *t* = 3.322; 24:2 P = 0.7925, *t* = 0.2750; cerebrosides—24:1 P = 0.0025, *t* = 4.975; 24:0 P = 0.0522, *t* = 2.374; 22:0 P = 0.0206, *t* = 3.121; 18:0 P < 0.0001, *t* = 9.396; 24:2 P = 0.9254, *t* = 0.09762; 20:0 P = 0.0090, *t* = 3.798; 22:1 P = 0.5524, *t* = 0.6293; 16:0 P = 0.0342, *t* = 2.730; sphingomyelin—24:1 P = 0.0116, *t* = 3.584; 24:0 P = 0.0040, *t* = 4.527; 24:2 P = 0.1726, *t* = 1.548; 18:0 P = 0.0033, *t* = 4.707; 16:0 P = 0.0773, *t* = 2.129; 22:0 P = 0.0044, *t* = 4.432; 20:0 P = 0.0016, *t* = 5.473; 22:1 P = 0.1303, *t* = 1.752; 16:1 P = 0.3844, *t* = 0.9381; 14:0 P = 0.1875, *t* = 1.487; 18:1 P = 0.1058, *t* = 1.903; 20:1 P = 0.8166, *t* = 0.2423; PIs—38:4 P = 0.0416, *t* = 2.583; 36:1 P = 0.0354, *t* = 2.703; 40:6 P = 0.1290, *t* = 1.760; 36:2 P = 0.0179, *t* = 3.232; 36:4 P = 0.1149, *t* = 1.843; 38:5 P = 0.1431, *t* = 1.684; 38:6 P = 0.4832, *t* = 0.7471; PEs—36:2 P = 0.0702, *t* = 2.199; 34:1 P = 0.0414, *t* = 2.587; 38:4 P = 0.9570, *t* = 0.0563; 38:5 P = 0.1397, *t* = 1.702; 38:6 P = 0.4854, *t* = 0.7433; 40:6 P = 0.7055, *t* = 0.3965; 36:1 P = 0.2995, *t* = 1.135; 36:3 P = 0.6899, *t* = 0.4188; 36:4 P = 0.1016, *t* = 1.932; 40:4 P = 0.6673, *t* = 0.4518; PCs—34:1 P = 0.0419, *t* = 2.577; 36:1 P = 0.2728, *t* = 1.207; 36:2 P = 0.2714, *t* = 1.211; 32:0 P = 0.7655, *t* = 0.3121; 32:1 P = 0.3562, *t* = 0.9993; 34:2 P = 0.2545, *t* = 1.260; 30:0 P = 0.7716, *t* = 0.3037; 36:4 P = 0.3984, *t* = 0.9090; 42:1 P = 0.0182, *t* = 3.219; 36:3 P = 0.3024, *t* = 1.128); *, P < 0.05; **, P < 0.01; ***, P < 0.001. Bars represent mean ± SEM.

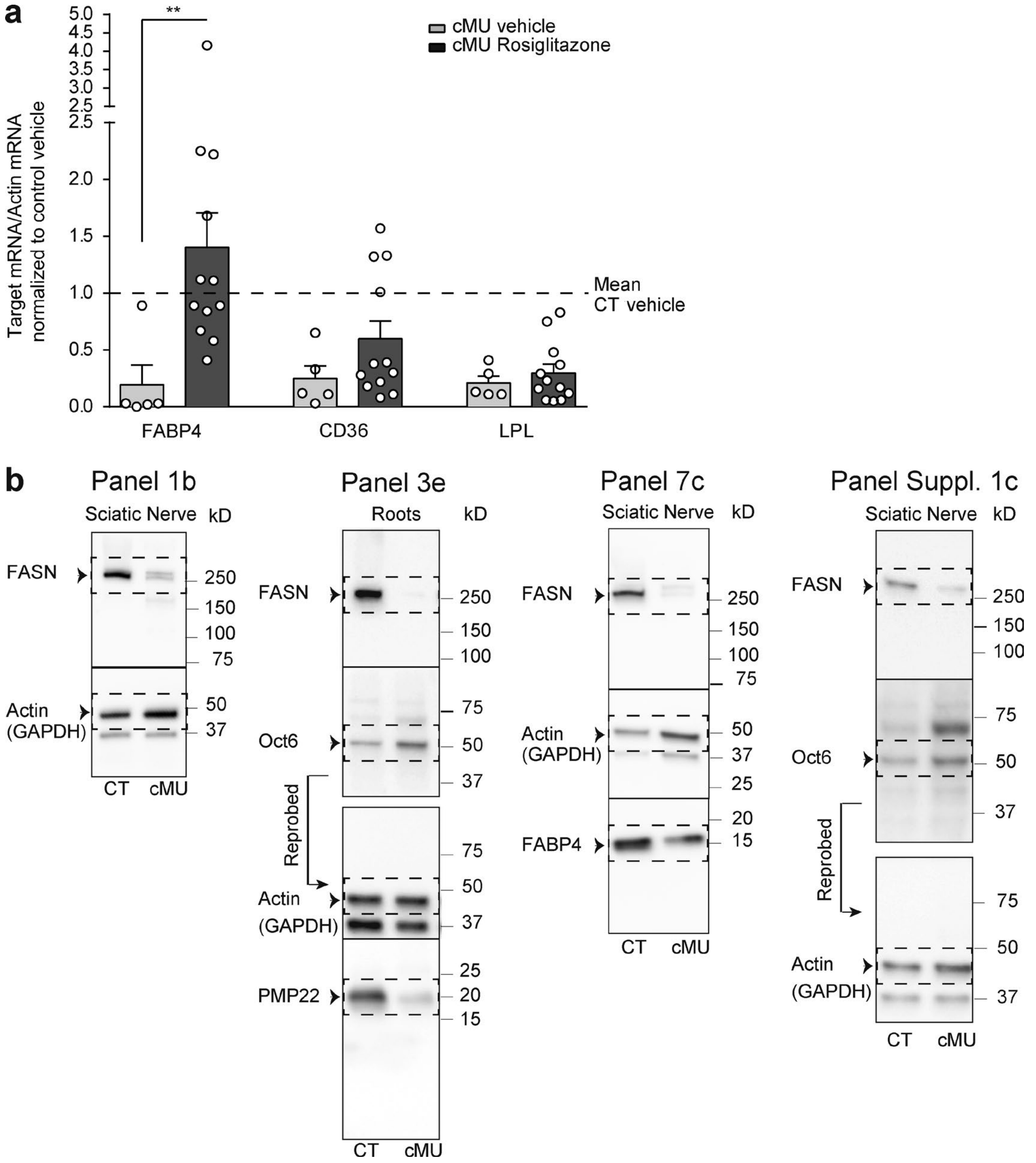

Figure S4. **Expression of PPARγ targets after rosiglitazone treatment (related to Fig. 8) and full-length immunoblots (related to Figs. 1, 3, 7, and S1).** **(a)** Graph of qRT-PCR analysis of FABP4, CD36, and LPL in sciatic nerves of P10 mutant (cMU) mice after vehicle or rosiglitazone treatment. Data normalized to β-actin and to the mean of control (CT) vehicle-injected. Data points represent $n = 5$ mice for cMU vehicle-injected, $n = 12$ mice for cMU rosiglitazone-injected (unpaired two-tailed Mann-Whitney test, FABP4 P = 0.006, $U = 5$; CD36 P = 0.2023 $U = 17.5$; LPL P = 0.7426 $U = 26.5$). **, P < 0.01. Bars represent mean ± SEM. **(b)** Full-length immunoblots. Cropped regions displayed in main figures are highlighted (dashed boxes). After transfer, membranes were cut as follows: Fig. 1 b, two pieces, cut below 75 kD; Fig. 3 e, three pieces, cut below 100 kD and 37 kD; Fig. 7 c, three pieces, cut below 75 kD and 25 kD; and Fig. S1 c, two pieces, cut below 100 kD. Gels were used as 4–12% Nu-PAGE precasted gradient gels (Fig. 3 e and Fig. S1 c) and 12% SDS-PAGE gels (Fig. 1 b and Fig. 7 c). Numbers refer to the molecular mass size markers (in kilodaltons).

Table S1. **Fatty acids and cholesterol content (expressed as percentage values) in the administered high fat (HFD) and standard (STD) diets**

Fat species	Fatty acid Nomenclature	Amount present in HFD (KLIBA NAFAG, 2127)	Amount presentin STD (KLIBA NAFAG, 3437)
		%	%
Butyric acid	C4	0.000	0.000
Caproic acid	C6	0.000	0.000
Caprylic acid	C8	0.000	0.000
Capric acid	C10	0.000	0.000
Lauric acid	C12	0.030	0.002
Myristic acid	C14	0.440	0.008
Pentadecanoic acid	C15	0.000	0.002
Palmitic acid	C16	8.220	0.719
Margaric acid	C17	0.000	0.004
Stearic acid	C18	4.500	0.157
Arachidic acid	C20	0.020	0.010
Behenic acid	C22	0.000	0.004
Lignoceric acid	C24	0.000	0.012
Myristoleic acid	C14:1	0.170	0.001
Palmitoleic acid	C16:1c7	1.000	0.052
Oleic acid	C18:1c9	14.120	1.024
Eicosenic acid	C20:1c9	0.000	0.013
Erucic acid	C22:1c9	0.000	0.002
Linoleic acid	C18:2c9c12	4.810	2.107
Linolenic acid	C18:3c9c12c15	0.590	0.212
Octadecatetraenoic acid	C18:4 (n-3)	0.000	0.000
Arachidonic acid	C20:4 (n-6)	0.560	0.009
Eicosapentanoic acid	C20:5 (n-3)	0.000	0.000
Clupanodonic acid	C22:5 (n-3)	0.000	0.001
Docosahexanoic acid	C22:6 (n-3)	0.000	0.001
Cholesterol		0.030	0.030

Table S2. **qRT-PCR probes and primers**

Probe	Forward primer (5′–3′)	Reverse primer (5′–3′)
β-actin	TTCTTTGCAGCTCCTTCGTT	ATGGAGGGGAATACAGCCC
CD36	AGATGACGTGGCAAAGAACAG	CCTTGGCTAGATAACGAACTCTG
FABP4	GGGAACCTGGAAGCTTGTCT	ACTCTCTGACCGGATGGTGA
Fasn	GTTGGCCCAGAACTCCTGTA	GTCGTCTGCCTCCAGAGC
LPL	ATGGATGGACGGTAACGGGAA	CCGATACAACCAGTCTACTACA
SCD1	CAGCCGAGCCTTGTAAGTTC	GCTCTACACCTGCCTCTTCG
SPTLC1	ACGAGGCTCCAGCATACCAT	TCAGAACGCTCCTGCAACTTG

Provided online is Table S3 in Excel. Table S3 shows regulated transcripts (ANOVA with Benjamini-Hochberg false discovery rate for multiple testing correction, $P < 0.05$, ≥1.5-fold change as up- or down-regulation) in sciatic nerves of P60 mutant mice compared with control mice identified by Affymetrix Transcriptome Analysis.
